# Machine learning driven prediction of cerebrospinal fluid rhinorrhoea following endonasal skull base surgery: A multicentre prospective observational study

**DOI:** 10.3389/fonc.2023.1046519

**Published:** 2023-03-23

**Keywords:** cerebrospinal fluid leak, cerebrospinal fluid rhinorrhoea, CSF, endoscopic endonasal, skull base surgery, machine learning - ML, neural network, outcome prediction

## Abstract

**Background:**

Cerebrospinal fluid rhinorrhoea (CSFR) is a common complication following endonasal skull base surgery, a technique that is fundamental to the treatment of pituitary adenomas and many other skull base tumours. The CRANIAL study explored CSFR incidence and related risk factors, particularly skull base repair techniques, *via* a multicentre prospective observational study. We sought to use machine learning to leverage this complex multicentre dataset for CSFR prediction and risk factor analysis.

**Methods:**

A dataset of 865 cases - 725 transsphenoidal approach (TSA) and 140 expanded endonasal approach (EEA) - with cerebrospinal fluid rhinorrhoea as the primary outcome, was used. Relevant variables were extracted from the data, and prediction variables were divided into two categories, preoperative risk factors; and repair techniques, with 6 and 11 variables respectively. Three types of machine learning models were developed in order to predict CSFR: logistic regression (LR); decision tree (DT); and neural network (NN). Models were validated using 5-fold cross-validation, compared *via* their area under the curve (AUC) evaluation metric, and key prediction variables were identified using their Shapley additive explanations (SHAP) score.

**Results:**

CSFR rates were 3.9% (28/725) for the transsphenoidal approach and 7.1% (10/140) for the expanded endonasal approach. NNs outperformed LR and DT for CSFR prediction, with a mean AUC of 0.80 (0.70-0.90) for TSA and 0.78 (0.60-0.96) for EEA, when all risk factor and intraoperative repair data were integrated into the model. The presence of intraoperative CSF leak was the most prominent risk factor for CSFR. Elevated BMI and revision surgery were also associated with CSFR for the transsphenoidal approach. CSF diversion and gasket sealing appear to be strong predictors of the absence of CSFR for both approaches.

**Conclusion:**

Neural networks are effective at predicting CSFR and uncovering key CSFR predictors in patients following endonasal skull base surgery, outperforming traditional statistical methods. These models will be improved further with larger and more granular datasets, improved NN architecture, and external validation. In the future, such predictive models could be used to assist surgical decision-making and support more individualised patient counselling.

## 1 Introduction

Endonasal operative approaches, including the transsphenoidal approach (TSA) and the expanded endonasal approach (EEA), have become workhorses in skull base neurosurgery (1, 2). They are predominately used in the treatment of pituitary adenomas and other sella-region neoplastic pathologies, with growing indications as these techniques evolve (3, 4). Despite the benefits the approaches offer in terms of access, the most common surgical complication remains cerebrospinal fluid rhinorrhoea (CSFR) – generally up to 5% in TSA and 20% in EEA, although these rates vary significantly across the literature (3, 5–18). CSFR has potentially serious sequelae, including meningitis; severe headache, pneumocephalus; increased length of hospital stays; re-admission; and need for further surgery (9, 12, 13).

Numerous risk factors have been identified for CSFR, including the presence of intraoperative cerebrospinal fluid (CSF) leak; revision surgery; and high body mass index (BMI) (19). A particularly important factor is the choice of skull base repair technique used intraoperatively (7, 10, 13, 16, 20). A recent expert consensus conducted *via* The Pituitary Society highlighted the practice variations across TSA, particularly during the skull base closure phase (21). A systematic review of the literature has found absolute heterogeneity across studies and centres in terms of skull base repair techniques, likely due to a lack of high-level comparative evidence (10).

CRANIAL (CSF rhinorrhoea after endonasal intervention to the skull base) was a prospective, multicentre observational study seeking to determine the: (1) scope of the methods of skull base repair; and (2) corresponding rates of CSFR (22–25). It represents the largest dataset of its kind, seeking to audit practice across the UK and Ireland. It revealed a CSFR incidence rate of 3.9% for TSA and 7.1% for EEA, lower than the literature standard, with minimal influence of particular repair regimes on CSFR incidence *via* traditional statistical analysis (25). In neurosurgery, machine learning models (MLs), or more specifically neural network models (NNs), have been shown to outperform these traditional statistical methods by leveraging their ability to utilise complex non-linear relationships between the various prediction variables (26–28). For example, NNs were able to identify the risk factors associated with a high risk of intraoperative CSF leak where traditional statistical analysis failed (29).

In this study, we use NNs on the granular multicentre CRANIAL dataset for analysis of CSFR, its risk factors, and the comparative effectiveness of skull base repair techniques in both TSA and EEA.

## 2 Methods

The transparent reporting of a multivariable prediction model for individual prognosis or diagnosis (TRIPOD) guided this methodology and report (30).

### 2.1 Data

#### 2.1.1 Collection

A detailed description of the generation of the CRANIAL dataset is described in (25). In brief, it is a multicentre dataset (30 centres in the UK and Ireland), collected *via* a prospective observational study in 3 phases encompassing November 2019 – July 2020 (22–25). All TSA (defined as transsphenoidal access to the sella alone) and EEA [defined as acquiring surgical access to an area beyond the sella (17, 19)] were included. The dataset is composed of baseline characteristic data (e.g., age; sex; tumour diameter), operative data (e.g., intraoperative CSF leak presence; skull base repair method) and postoperative outcomes (e.g., CSFR) (22–25). A taxonomy for skull base repair was adapted from a systematic review of the literature (10, 24). Postoperative CSFR was confirmed biochemically and/or required intervention (CSF diversion and/or operative repair) (22–25).

#### 2.1.2 Processing

The dataset contained 866 participants (726 TSA, 140 EEA). Variables relevant to CSFR (as guided by consensus-derived protocol and literature review) were extracted from the dataset (24, 25). The primary outcome was CSFR. Prediction variables (predictors) were divided into two prediction categories: ‘preoperative risk factors for CSFR’ (risk factors) and ‘repair techniques used’ (repair techniques), with 6 and 11 predictors respectively, as shown in **Table 1**. Tumour type has been excluded as a risk factor predictor in this study, as many of the tumour types are too few in number for internal validation. Ultimately, this results in three prediction categories: 1) risk factors; 2) repair technique; 3) risk factors and repair technique.

**Table 1:** Distribution details of variables (predictors, approach, outcome) split by approach categories.

Category	Parameter	Distribution		
**Approach**	*Surgical Approach*	TSA 725 (83.4%)	EEA 140 (16.2%)	TSA or EEA (866)
**Risk Factors**	*Median Age (IQR)*	53 (41-64) years	51 (34-62) years	53 (40-63) years
	*Male Sex*	355 (49.0%)	61 (43.5%)	416 (48.0%)
	*BMI > 30*	210 (29.0%)	28 (20.0%)	238 (27.5%)
	*Tumour Diameter ≥ 1cm*	606 (83.6%)	131 (93.6%)	737 (85.2%)
	*Revision Surgery*	98 (13.5%)	21 (15.0%)	119 (13.8%)
	*Presence of Intraoperative CSF Leak*	214 (29.5%)	79 (56.4%)	293 (33.9%)
**Repair Techniques**	*CSF Diversion*	29 (4.0%)	38 (27.1%)	67 (7.8%)
	*Dural Closure*	0 (0.0%)	0 (0.0%)	0 (0.0%)
	*Dural Replacement*	196 (27.0%)	66 (47.1%)	262 (30.3%)
	*Vascularised Flap*	116 (16.0%)	90 (64.3%)	206 (23.8%)
	*Tissue Graft*	221 (30.5%)	65 (46.4%)	286 (33.1%)
	*Synthetic Graft*	203 (28.0%)	47 (33.6%)	251 (28.9%)
	*Tissue Glue*	473 (65.2%)	114 (81.4%)	587 (67.7%)
	*Haemostatic Agent*	439 (60.6%)	93 (66.4%)	532 (61.5%)
	*Rigid Buttress*	31 (4.3%)	17 (12.1%)	48 (5.5%)
	*Gasket Seal*	15 (2.1%)	11 (7.9%)	26 (3.0%)
	*Nasal Packing*	518 (71.4%)	116 (82.9%)	635 (73.3%)
**Outcome**	*CSFR*	28 (3.9%)	10 (7.1%)	38 (4.4%)

All variables are binary, excluding age which is continuous. For the binary variables the number of entries where the variable is present (represented as a 1) is given, with the round brackets giving the percentage (%) proportion. For the singular continuous parameter (age), median; and inter-quartile range (IQR) are given instead.

The participants were divided into three approach categories: TSA; EEA; TSA or EEA. This, therefore, leads to nine total subcategories for each method: a separate model for the three approach categories multiplied by the three prediction categories. One additional model was created using surgical approach as a predictor, and hence the final number of subcategories for each method is 10.

Binary values (1 for used, 0 for not used) were set for all 11 repair technique predictors, and if missing, assumed not to be used and hence set to 0. Binary values were also set for the risk factor predictors: sex (male set to 1, female set to 0); BMI (>30 set to 1, ≤ 30 set to 0); tumour size (tumour diameter ≥ 1cm set to 1, tumour diameter < 1cm set to 0); intraoperative CSF leak (grade 1, 2, 3, or present but unknown grade set to 1, not present set to 0). Intraoperative CSF leak grade was not set as a categoric variable as conversion to a nominal variable would split each grade into its own prediction variable, leading to poorer correlations; and conversion to an ordinal variable would require the loss of the present but unknown grade category, representing an 18% loss of positive cases. Age was left as a continuous predictor but normalised to a Gaussian distribution with mean 0 and standard deviation 1. If any risk factor predictor was missing, the participant was excluded. Binary values were also assigned to the surgical approach (TSA set to 0, EEA set to 1) and CSFR (1 for present, 0 for not present), and if either was missing, the participant was excluded.

### 2.2 Model development

#### 2.2.1 Machine learning

Three ML methods have been used in this study: logistic regression models (LRs); decision tree models (DTs); and neural network models. These have been chosen as they represent the increasing complexity of ML methods as measured by the number of adjustable parameters present in each model. The code is written in Python 3.8 (31, 32).

#### 2.2.2 Validation

**Figure 1:**
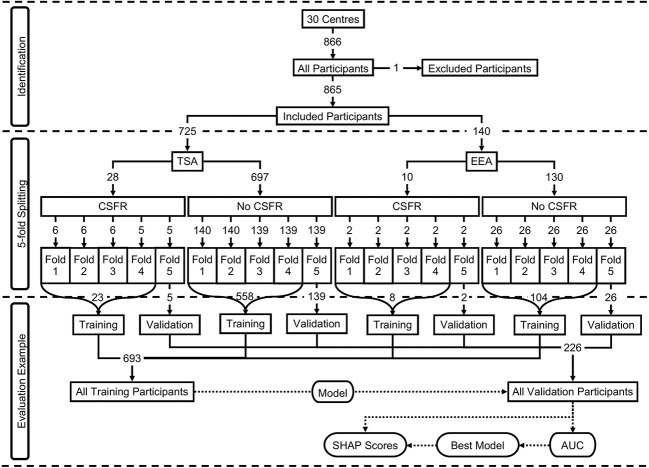
Participants breakdown displayed as a flowchart. The top section (identification) displays the included and excluded participants. The middle section (5-fold splitting) displays how the 5-folds were created, including the breakdown by surgical approach and outcome. The predictor distributions of the overall participants can be seen in **Table 1**, and the predicter distributions for each of the 5-folds can be seen in **Supplementary Material Table 3** The bottom section (evaluation example) displays an example of a model training on one fold’s training dataset, and then evaluated on the same fold’s validation dataset.

For validation, 5-fold cross-validation was used, with an 80:20 training to validation split for each fold. This was achieved by first separating the participants by the two surgical approaches, and then further separating the participants by the two CSFR outcomes, leading to four subgroups of participants (TSA with CSFR; TSA without CSFR; EEA with CSFR; EEA without CSFR). For each of these subgroups, the participants were randomly split into 5-folds, and assigned an appropriate fold number (1 to 5). Next, the participants from each output subgroup were combined by fold number, producing two groups separated by surgical approach. Finally, these two approach groups were combined by fold number. This means there are three groups separated by approach (TSA; EEA; TSA or EEA), where the ratio between the two CSFR binary outputs remains approximately the same for each fold as found in the data. Moreover, the ratio between TSA and EEA in the ‘TSA or EEA’ approach group remains the same as found in the data. This group methodology is displayed in **Figure 1** and variable (predictors, surgical approach, outcome) distributions for each of the 5-folds can be found in **Supplementary Material Table 3**.

For each fold, after a model was trained on the other folds’ participants (training dataset), it was then evaluated on the fold participants (validation dataset), and the evaluation metrics recorded. After repeating this for all folds, the evaluation metrics for both the mean-average and standard deviations were calculated across the 5-folds. Hyperparameter tuning of all MLs were performed through multiple runs on the validation dataset *via* grid search, and for NNs this was done at the epoch level.

Given the number of participants with CSFR represents just 4.4% of the data, for the training dataset, these participants were oversampled randomly such that they represent 50% of the data. This prevents overfitting to the entries without CSFR, where the models would simply always predict CSFR not occurring, leading to an effectively useless model. For evaluation metric calculations of both the training and validation datasets, no such oversampling was done.

#### 2.3 Evaluation

##### 2.3.1 AUC

The primary evaluation metric to compare MLs is the ‘area under the receiver operating characteristic’ (AUC) curve, which ensures a balance of both the sensitivity (true positive rate) and specificity (true negative rate), and these two are also given as secondary evaluation metrics.

##### 2.3.2 SHAP

To compare a specific predictor’s contribution to a NN predicting CSFR, ‘Shapley additive explanations’ (SHAP) scores were calculated. The SHAP method does this by calculating all possible combinations of the predictors, inputting each predictor combination into the model, and evaluating the combination’s contribution to the model on the validation dataset. By doing this, each predictor’s contribution to the model is calculated in isolation of the other predictors while also accounting for the non-linear relationships (33).

The magnitude (independent of score sign) of a SHAP score determines how large of a contribution that predictor has to the NN’s outcome prediction. The sign of a predictor’s score determines whether the NN has an increased (if positive) or decreased (if negative) probability of predicting a CSFR. A red dot means this probability is due to the predictor being present, a blue dot means it is due to the predictor not being present. If the red and blue dots have a clear boundary about a score of 0.0 and are not overlapping, this is interpreted as the predictor’s value being highly correlated with the NN’s outcome prediction. Similarly, the greater the overlap, the weaker the correlation. (Note purple dots are seen for age as it is a continuous variable: here red represents the oldest participant; blue the youngest participant; and purple for the ages in between.)

## 3 Results

### 3.1 Data

Out of the initial 866 participants, one case was removed due to missing age, resulting in 855 cases (725 TSA, 140 EEA). Full distribution details of all included variables (predictors, surgical approach, outcome) are given in **Table 1**, and the distribution across each of the 5-folds is given in **Supplementary Material Table 3**.

### 3.2 Machine learning

The trained models, and a guide on how to use them, can be found in an open-access code repository (32).

#### 3.2.1 Logistic regression

The LRs were created using scikit-learn 0.23.2 (34), and liblinear was chosen as the optimisation algorithm. The inverse of regularisation strength (C-value) was chosen as a hyperparameter to be tuned, and found to have an optimal value of 0.1, with the remaining parameters set as default values as stated in (35).

#### 3.2.2 Decision tree

The DTs were created using scikit-learn 0.23.2 (34), and ‘classification and regression trees’ (CART) was chosen as the tree algorithm. The maximum tree depth was chosen as a hyperparameter to be tuned, and found to have an optimal value of 4, with the remaining parameters set as default values as stated in (36).

#### 3.2.3 Neural network

The NNs were created using PyTorch 1.8.1 (37) and run on an Nvidia 2070 Super GPU using CUDA 11.2. A feedforward network was created with a linear input layer of 8 neurons, 3 linear hidden layers with 12 neurons each, and a final linear output layer with one neuron, followed by a sigmoid activation function with a 0.5 threshold for CSFR classification. For the non-output layers, the ‘rectified linear activation unit’ (ReLu) was used as the activation function, with a 0.35 dropout. Binary cross-entropy was used as the loss function and ‘stochastic gradient descent’ (SGD) was used as the optimiser, with learning rate; momentum; batch size; and number of epochs hyperparameters to be tuned. A learning rate of 0.001; momentum of 0.9; batch size of 100; and number of epochs equalling 100 were found to be optimal.

### 3.3 Evaluation

#### 3.3.1 AUC

**Figure 2:**
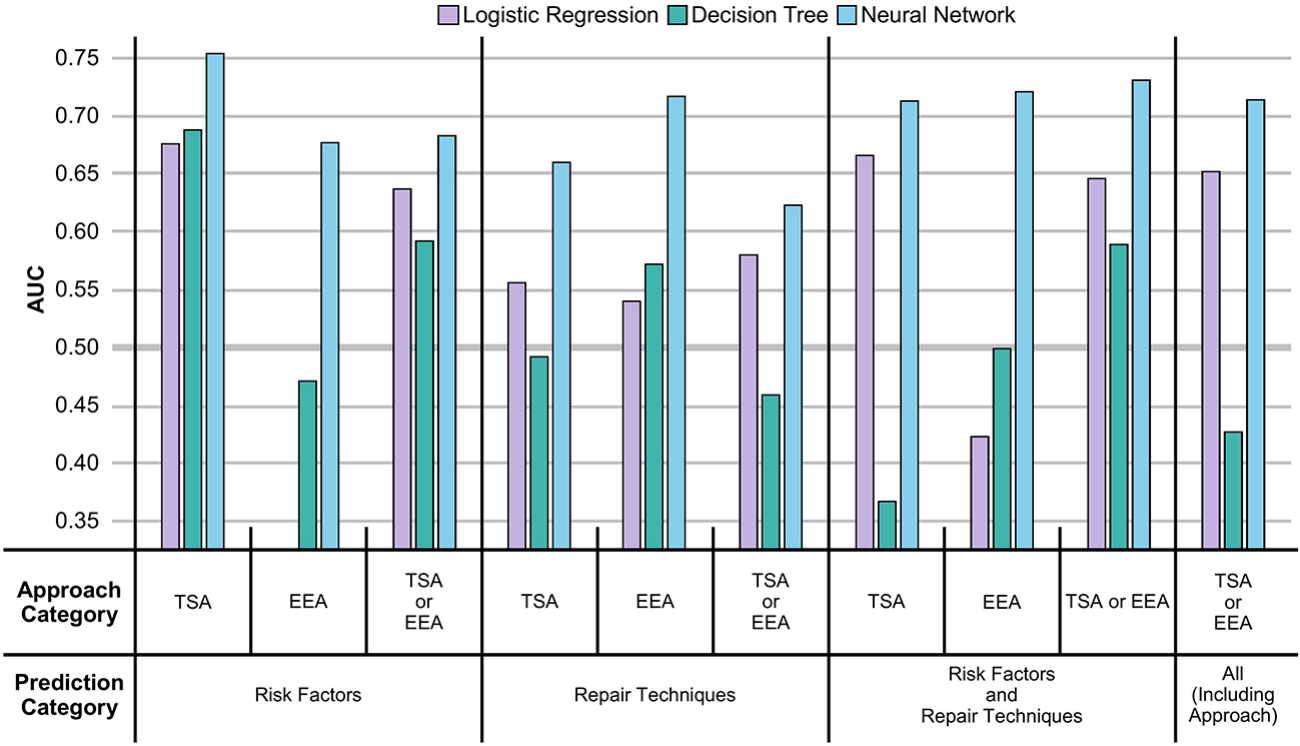
AUC of MLs displayed as a vertical bar chart. The AUC scale ranges from 0.35 to 0.75, with a thicker line at 0.50. Error bars representing the standard deviation across the 5-folds are not given. The AUC for LRs in the risk factors EEA case is not displayed as the AUC (0.22) is too low. The full values, including the standard deviation error bars, can be seen in **Table 2**.

From **Figure 2** and **Table 2**, it can be seen that the NNs were able to predict the existence of CSFR across all prediction categories and approach categories with an AUC > 0.50 (an AUC of 0.50 is equivalent to a model that randomly predicts CSFR). Both LRs and DTs performances are outperformed by NNs, and for a few instances have an AUC < 0.5.

**Table 2:** Performance of MLs.

Predictor Category	Surgical Category	ML Method	Training			Validation		
			AUC	Sensitivity	Specificity	AUC	Sensitivity	Specificity
**Risk Factors**	TSA	LR	0.74±0.02	0.75±0.02	0.64±0.02	0.68±0.10	0.64±0.11	0.63±0.04
		DT	0.79±0.02	0.63±0.04	**0.79±0.01**	0.69±0.18	0.56±0.21	**0.79±0.02**
		NN	**0.83±0.02**	**0.78±0.12**	0.71±0.19	**0.75±0.08**	**0.69±0.17**	0.70±0.18
	EEA	LR	0.62±0.02	0.75±0.08	0.38±0.05	0.22±0.09	0.30±0.24	0.40±0.15
		DT	**0.87±0.05**	**0.93±0.10**	**0.68±0.12**	0.47±0.18	0.30±0.40	**0.62±0.06**
		NN	0.59±0.10	0.80±0.40	0.30±0.37	**0.68±0.08**	**0.80±0.40**	0.28±0.37
	TSA or EEA	LR	0.69±0.02	**0.69±0.05**	0.61±0.02	0.64±0.11	**0.65±0.19**	0.61±0.04
		DT	**0.83±0.01**	0.65±0.04	**0.83±0.04**	0.59±0.12	0.36±0.17	**0.81±0.03**
		NN	0.79±0.03	0.63±0.17	0.78±0.12	**0.68±0.08**	0.45±0.19	0.76±0.12
**Repair Techniques**	TSA	LR	0.68±0.04	0.62±0.07	0.61±0.09	0.56±0.14	0.43±0.26	0.59±0.09
		DT	**0.91±0.05**	**0.93±0.15**	**0.78±0.15**	0.49±0.22	0.10±0.20	**0.74±0.13**
		NN	0.74±0.08	0.73±0.20	0.61±0.23	**0.66±0.08**	**0.60±0.21**	0.61±0.23
	EEA	LR	**0.81±0.05**	**0.80±0.10**	0.64±0.07	0.54±0.16	0.40±0.37	0.56±0.12
		DT	0.79±0.04	0.75±0.03	**0.69±0.05**	0.57±0.06	0.53±0.13	**0.67±0.05**
		NN	0.76±0.11	0.75±0.39	0.59±0.29	**0.72±0.14**	**0.70±0.40**	0.50±0.37
	TSA or EEA	LR	0.69±0.01	0.70±0.04	0.59±0.04	0.58±0.06	**0.50±0.16**	0.59±0.04
		DT	**0.77±0.04**	**0.73±0.15**	0.68±0.08	0.46±0.07	0.35±0.25	0.68±0.12
		NN	0.77±0.05	0.72±0.20	**0.70±0.17**	**0.62±0.05**	0.49±0.22	**0.69±0.17**
**Risk Factors and Repair Techniques**	TSA	LR	0.79±0.01	0.73±0.04	0.68±0.01	0.67±0.09	**0.49±0.25**	0.67±0.06
		DT	0.86±0.04	0.88±0.08	0.72±0.05	0.37±0.16	0.20±0.24	0.67±0.11
		NN	**0.90±0.05**	**0.89±0.16**	**0.80±0.13**	**0.71±0.09**	0.49±0.29	**0.80±0.10**
	EEA	LR	**0.81±0.03**	**0.85±0.09**	0.59±0.05	0.42±0.20	0.40±0.37	0.47±0.07
		DT	0.75±0.02	0.76±0.07	0.61±0.06	0.50±0.07	0.41±0.10	0.59±0.05
		NN	0.79±0.10	0.58±0.38	**0.80±0.18**	**0.72±0.09**	**0.50±0.45**	**0.78±0.18**
	TSA or EEA	LR	0.75±0.01	0.70±0.04	0.66±0.01	0.65±0.10	0.57±0.21	0.64±0.04
		DT	0.84±0.01	0.78±0.15	**0.73±0.15**	0.59±0.13	0.47±0.17	**0.70±0.16**
		NN	**0.88±0.05**	**0.91±0.14**	0.67±0.17	**0.73±0.03**	**0.63±0.31**	0.64±0.17
**All (Including Approach)**	TSA or EEA	LR	0.76±0.01	0.73±0.03	0.67±0.01	0.65±0.09	**0.59±0.24**	0.65±0.03
		DT	0.91±0.03	**1.00±0.00**	**0.74±0.07**	0.43±0.19	0.20±0.40	0.67±0.07
		NN	**0.91±0.02**	0.96±0.06	0.69±0.06	**0.71±0.06**	0.57±0.13	**0.68±0.06**

Values are given to two decimal places in the form ‘mean ± standard deviation’ calculated over the 5-fold cross-validation. Bolded values highlight the best performing metric in the (subset, approach) category for that column’s performance metric.

Comparing approach categories, it can be seen all three categories have similar NNs performances, but EEA performs worse than TSA for LRs. After mean-averaging across approach categories, and then comparing NNs performance across prediction categories, it can be seen risk factors slightly outperform repair techniques, which are in turn outperformed when all predictors (excluding surgical approach) are used. The inclusion of surgical approach as a predictor does not improve NN performance.

As seen in **Table 2**, a high AUC in the training dataset does not necessarily correspond to a high AUC in the validation dataset. In particular, for DTs, the issue is exacerbated. For example, for the TSA repair techniques, a 0.86 training AUC translates to a 0.37 validation AUC for DT, compared to a 0.79 training AUC to 0.67 validation AUC translation for LR, or 0.90 training AUC to 0.71 validation AUC translation for NN.

#### 3.3.2 SHAP

**Figure 3:**
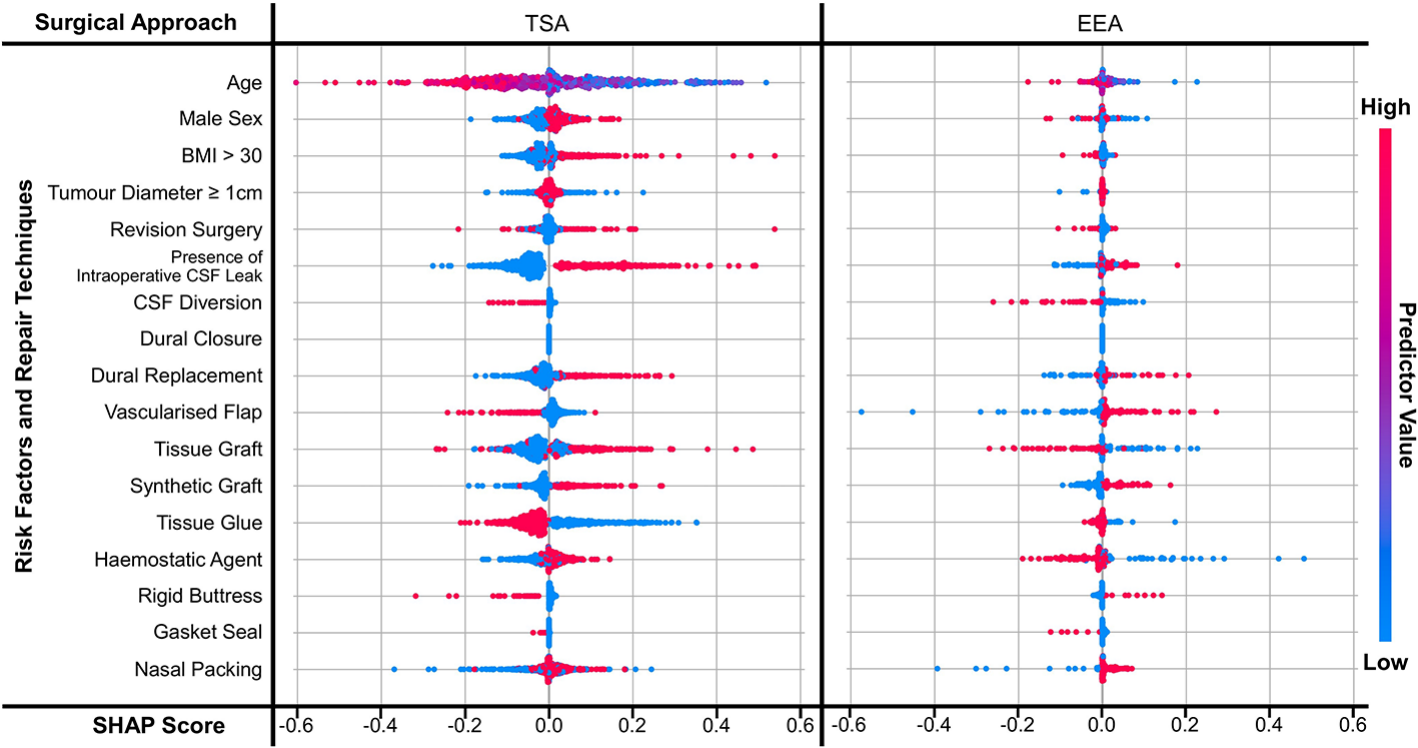
SHAP scores for predictors displayed as a bee diagram for the predictor category ‘risk factors and repair techniques’, where the NNs are split by approach. Scores are shown for each predictor across all 5-folds. As shown in the ‘predictor value’ legend – a high value is indicated in red, and a low value is indicated by blue; for binary variables this means red indicates a value of 1 (i.e. present) and blue indicates a value of 0 (i.e. not present).

**Figure 3** displays the SHAP scores for each predictor for two NNs (TSA risk factors and repair techniques; EEA risk factors and repair techniques). **Supplementary Figures 1**, **2** display the SHAP scores for the remaining eight NNs and **Supplementary Table 2** shows the SHAP correlation coefficients for all ten NNs - consistent with the trends shown in **Figure 3**. Comparing approach categories, the SHAP scores are larger in magnitude for TSA than EEA. Comparing prediction categories, the SHAP scores for risk factors have a clearer boundary between not present and present than repair techniques.

Focusing on TSA risk factors, the presence of intraoperative CSF leak appears to be the strongest predictor of CSFR within the NN (**Figure 3** and **Supplementary Table 4**). This is followed by younger age, elevated BMI, revision surgery, and male sex seem to also increase the probability of CSFR, albeit with a weaker correlation. EEA risk factors have a much smaller magnitude and weaker correlation, with intraoperative CSF leak having the strongest relative relationship with CSFR incidence (**Figure 3** and **Supplementary Table 4**).

The impact of repair techniques on CSFR is less clear. In TSA, the use of CSF diversion, vascularised flaps, rigid buttresses +/- gasket sealing, and tissue glues appear to be protective against CSFR (**Figure 3** and **Supplementary Table 4**). However, synthetic grafts, and to a lesser extent, dural replacement and tissue grafts appear to be associated with CSFR occurrence. For EEA, CSF diversion, gasket sealing, and to a lesser extent tissue grafts and haemostatic agents appear to reduce CSFR incidence. Synthetic grafts, vascularised flaps and dural replacement appear to be associated with CSFR occurrence.

## 4 Discussion

### 4.1 Principal findings

In this study, three ML methods were applied to a complex, multicentre, prospective skull base neurosurgery database encompassing CSFR and relevant predictor data (risk factors and intraoperative repair techniques).

Firstly, NNs outperformed LR and DT for CSFR prediction, with a mean AUC of 0.80 (0.70-0.90) for TSA and 0.78 (0.60-0.96) in EEA, when all risk factor and intraoperative repair data were integrated into the model. This is likely explained by NNs’ known ability to learn complex non-linear relationships, even in the context of a large number of variables (27, 28). In this dataset, this likely reflects the use of multiple repair techniques synergistically and in layers, tailored to risk factors encountered on a case-by-case basis (10). NNs achieved this despite the class imbalance caused by a CSFR rate lower than the literature standard, with oversampling 5-fold validation (25, 28). Furthermore, there was an iterative improvement in NN performance with larger datasets, with TSA models (725 cases) generally outperforming EEA models (140 cases), and the use of risk factor data with intraoperative repair technique data improved CSFR prediction when compared with using a single data category.

Using SHAP scores, the relationship between predictor variables (risk factors and intraoperative repair techniques) was explored for their relative predictive value within NN models. The presence of intraoperative CSF leak was the most prominent risk factor for CSFR in TSA and EEA, which is in line with existing studies (7, 10, 20, 38, 39). The presence of elevated BMI and revision surgery were also associated with CSFR for the larger TSA dataset, again reflected in the literature (16). Modern repair regimes are tailored to risk factors, and this analysis consolidates pertinent factors to guide surgeons in repair technique decision-making (10).

When compared with traditional statistical models (e.g., multivariate logistic regression models), which suggested tissue glues alone may have a benefit in TSA, NN SHAP analysis has highlighted new potential relationships within the dataset, as well as reproducing the potential impact of tissue glues on CSFR rates (25). Specifically, CSF diversion and gasket sealing appear to be strong predictors of the absence of CSFR in both TSA and EEA – in line with RCT evidence (lumbar drainage) and numerous institutional series (gasket sealing) (10, 20, 40–42). Synthetic grafts and dural replacements (which often have overlapping materials) were associated with the development of CSFR in both TSA and EEA. Whilst autologous tissue repair had contradictory results depending on approach nasoseptal flaps (associated with CSFR in EEA, but protective against CSFR in TSA) and tissue grafts (associated with CSFR in TSA, but protective against CSFR in EEA). The reasons for this are difficult to further ascertain within the NN structure, but theoretically may be due to differences in the groups of patients undergoing these repairs (for example, patients deemed at higher risk of CSFR at a baseline in EEA undergo nasoseptal flap) (25).

### 4.2 Comparison to literature

To our knowledge, only one other study has applied ML to a similar research question. However, this study examined intraoperative CSF leak (rather than postoperative CSFR), had a more imaging-centric dataset, was single centre (rather than 30 centres), and resultantly smaller volume (154 vs 855 cases) (29). Using a NN, Staartjes et al. were able to identify risk factors (higher Hardy grade, revision surgery, older age) whereas conventional statistical methods were unable to do so, echoing our experience in this study (29). There are however numerous studies utilising traditional statistical techniques in institutional case series in this field. Patel et al. use logistic regression models in a large volume single centre series, finding elevated BMI and hydrocephalus as significant risk factors for CSFR (43). Hannan et al. used similar methods and found that surgical experience, intraoperative CSF leak, Cushing’s disease and the absence of nasoseptal flap use as CSFR risk factors (38). Similarly, Xue et al. highlighted intraoperative CSF leak as a key CSFR predictor and recommend nasoseptal flaps and lumbar drainage to decrease its incidence (39). Finally, Cai used a Least Absolute Shrinkage and Selection Operator (LASSO) model with multivariate logistic regression in a single centre moderate volume data set in the context of intraoperative CSF leak prediction, suggesting tumour size and preoperative albumin as key determinants (44).

### 4.3 Strengths and limitations

One of the strengths of this study is the large number of centres the data has come from, leading to data diversity, and hence improving the generalisability of the models. Overfitting was mitigated against in NNs using drop-out between layers, whilst evidence of this remained in LR and DT models (mismatch between training and validation datasets). More data (with more CSFR cases), from more countries, and an external validation dataset would be useful to improve model performance and generalisability further. Moreover, although our study is prospective with an internally validated dataset, observational studies inherently contain various types of bias, and so the correlations made may not be reflective of the overall population.

Another strength of the study is the large number and variety of predictors used, which improves model performance. On the other hand, the choice of predictors is also a limit, as other predictors, (such as type of tumour); or more granular versions of the predictors (such as intraoperative CSF leak grade rather than binary presence), have not been used. Furthermore, the range of ML models trialled, and the use of SHAP analysis, showing how and why NNs outperform LRs and DTs is a relative study strength. Nevertheless, the choice of NNs is limited to one simple architecture, and it is unknown whether more sophisticated architectures will improve performance in the future. Finally, this study shares the common issue of interpretability that many ML studies have, particularly the SHAP analysis, which may affect model usability and uptake by clinicians.

## 5 Conclusion

Three ML methods were applied to a complex, multicentre, prospective skull base neurosurgery database to predict CSFR following endonasal skull base surgery, and prediction variables that are most important for its development. NNs outperformed traditional statistical models and other ML models in CSFR prediction. NNs also uncovered relationships between risk factors and repair techniques on CSFR, which were otherwise not detected using traditional statistical approaches. These models will be improved further with larger and more granular datasets, improved NN architecture, and external validation. In the future, the next generation of these predictive models could be used to assist surgical decision-making and to support more individualised patient counselling.

## Data availability statement

The original contributions presented in the study are included in the article/**Supplementary Material**. Further inquiries can be directed to the corresponding author.

## Ethics statement

Formal institutional ethical board review and informed consent from human participants were not required owing to the nature of the study (seeking to evaluate local services as an observational study) and this was confirmed by the Health Research Authority, UK.

## CRANIAL Consortium

Adrito Das*†, Wellcome/EPSRC Centre for Interventional and Surgical Sciences, University College London, London; Danyal Z Khan*†, Department of Neurosurgery, National Hospital for Neurology and Neurosurgery, London; **Danail Stoyanov‡**, Wellcome/EPSRC Centre for Interventional and Surgical Sciences, University College London, London; **Hani J Marcus‡**, Department of Neurosurgery, National Hospital for Neurology and Neurosurgery, London; **Soham Bandyopadhyay**, Oxford University Global Surgery Group, Nuffield Department of Surgical Sciences, University of Oxford, Oxford; **Benjamin E Schroeder**, Department of Neurology, University Hospital of Wales, Cardiff University, Cardiff; **Vikesh Patel**, Division of Neurosurgery, Cambridge University Hospitals Trust, Cambridge; **Alice O’Donnell**, Birmingham Medical School, University of Birmingham, Birmingham; **Neurology and Neurosurgery Interest Group**, NANSIG; **British Neurosurgical Trainee Research Collaborative**, BNTRC; **Anastasios Giamouriadis**, Department of Neurosurgery, Aberdeen Royal Infirmary, Aberdeen; **Pragnesh Bhatt**, Department of Neurosurgery, Aberdeen Royal Infirmary, Aberdeen; **Bhaskar Ram**, Department of Otorhinolaryngology, Aberdeen Royal Infirmary, Aberdeen; **Adithya Varma**, Department of Neurosurgery, Aberdeen Royal Infirmary, Aberdeen; **Philip Weir**, Department of Neurosurgery, Royal Victoria Hospital, Belfast; **Brendan Hanna**, Department of Otorhinolaryngology, Royal Victoria Hospital, Belfast; **Theodore C Hirst**, Department of Neurosurgery, Royal Victoria Hospital, Belfast; **Patrick McAleavey**, Department of Neurosurgery, Royal Victoria Hospital, Belfast; **Alessandro Paluzzi**, Department of Neurosurgery, Queen Elizabeth Hospital Birmingham, Birmingham; **Georgios Tsermoulas**, Department of Neurosurgery, Queen Elizabeth Hospital Birmingham, Birmingham; **Shahzada Ahmed**, Department of Otorhinolaryngology, Queen Elizabeth Hospital Birmingham, Birmingham; **Wai Cheong Soon**, Department of Neurosurgery, Queen Elizabeth Hospital Birmingham, Birmingham; **Yasir Arafat Chowdhury**, Department of Neurosurgery, Queen Elizabeth Hospital Birmingham, Birmingham; **Suhaib Abualsaud**, Department of Neurosurgery, Queen Elizabeth Hospital Birmingham, Birmingham; **Shumail Mahmood**, Department of Neurosurgery, Queen Elizabeth Hospital Birmingham, Birmingham; **Paresh Naik**, Department of Otorhinolaryngology, Queen Elizabeth Hospital Birmingham, Birmingham; **Zohra Haiderkhan** Department of Neurosurgery, Queen Elizabeth Hospital Birmingham, Birmingham; **Rafid Al-Mahfoudh**, Department of Neurosurgery, Hurstwood Park Neurosciences Centre and Royal Sussex County Hospital, Brighton; **Andrea Perera**, Department of Neurosurgery, Hurstwood Park Neurosciences Centre and Royal Sussex County Hospital, Brighton; **Mircea Rus**, Department of Neurosurgery, Hurstwood Park Neurosciences Centre and Royal Sussex County Hospital, Brighton; **Adam Williams**, Department of Neurosurgery, Southmead Hospital Bristol, Bristol; **Charles Hand**, Department of Neurosurgery, Southmead Hospital Bristol, Bristol; **Kumar Abhinav**, Department of Neurosurgery, Southmead Hospital Bristol, Bristol; **Cristina Cernei**, Department of Neurosurgery, Southmead Hospital Bristol, Bristol; **Aiman Dilnawaz**, Department of Neurosurgery, Southmead Hospital Bristol, Bristol; **Richard Mannion**, Division of Neurosurgery, Cambridge University Hospitals Trust, Cambridge; **Thomas Santarius**, Division of Neurosurgery, Cambridge University Hospitals Trust, Cambridge; **James Tysome**, Division of Otorhinolaryngology, Cambridge University Hospitals Trust, Cambridge; **Rishi Sharma**, Division of Otorhinolaryngology, Cambridge University Hospitals Trust, Cambridge; **Angelos G Kolias**, Division of Neurosurgery, Cambridge University Hospitals Trust, Cambridge; **Neil Donnelly**, Division of Otorhinolaryngology, Cambridge University Hospitals Trust, Cambridge; **Vikesh Patel**, Division of Neurosurgery, Cambridge University Hospitals Trust, Cambridge; **Ashwin Venkatesh**, Division of Neurosurgery, Cambridge University Hospitals Trust, Cambridge; **Caroline Hayhurst**, Department of Neurosurgery, University Hospital of Wales, Cardiff; **Amr Mohamed**, Department of Neurosurgery, University Hospital of Wales, Cardiff; **Benjamin Stew**, Department of Otorhinolaryngology, University Hospital of Wales, Cardiff; **Joseph Merola**, Department of Neurosurgery, University Hospital of Wales, Cardiff; **Setthasorn Zhi Yang Ooi**, Department of Neurosurgery, University Hospital of Wales, Cardiff; **Mahmoud Kamel**, Department of Neurosurgery, Cork University Hospitals, Ireland; **Mohammad Habibullah Khan**, Department of Otorhinolaryngology, Cork University Hospitals, Ireland; **Sahibzada Abrar**, Department of Neurosurgery, Cork University Hospitals, Ireland; **Christopher Mckeon**, Department of Neurosurgery, Cork University Hospitals, Ireland; **Daniel McSweeney**, Department of Neurosurgery, Cork University Hospitals, Ireland; **Mohsen Javadpour**, Department of Neurosurgery, National Neurosurgical Centre, Beaumont Hospital, Ireland; **Peter Lacy**, Department of Otorhinolaryngology, National Neurosurgical Centre, Beaumont Hospital, Ireland; **Daniel Murray**, Department of Neurosurgery, National Neurosurgical Centre, Beaumont Hospital, Ireland; **Elena Roman**, Department of Neurosurgery, National Neurosurgical Centre, Beaumont Hospital, Ireland; **Kismet Hossain-Ibrahim**, Department of Neurosurgery, Ninewells Hospital, Dundee; **Peter Ross**, Department of Otorhinolaryngology, Ninewells Hospital, Dundee; **David Bennett**, Department of Neurosurgery, Ninewells Hospital, Dundee; **Nathan McSorley**, Department of Neurosurgery, Ninewells Hospital, Dundee; **Adam Hounat**, Department of Neurosurgery, Ninewells Hospital, Dundee; **Patrick Statham**, Department of Clinical Neurosciences, BioQuarter, Edinburgh; **Mark Hughes**, Department of Clinical Neurosciences, BioQuarter, Edinburgh; **Alhafidz Hamdan**, Department of Clinical Neurosciences, BioQuarter, Edinburgh; **Caroline Scott**, Department of Clinical Neurosciences, BioQuarter, Edinburgh; **Jigi Moudgil-Joshi**, Department of Clinical Neurosciences, BioQuarter, Edinburgh; **Anuj Bahl**, Department of Neurosurgery, Hull University Teaching Hospitals, Hull; **Anna Bjornson**, Department of Neurosurgery, Hull University Teaching Hospitals, Hull; **Daniel Gatt**, Department of Neurosurgery, Hull University Teaching Hospitals, Hull; **Nick Phillips**, Department of Neurosurgery, Leeds Teaching Hospitals, Leeds; **Neeraj Kalra**, Department of Neurosurgery, Leeds Teaching Hospitals, Leeds; **Melissa Bautista**, Department of Neurosurgery, Leeds Teaching Hospitals, Leeds; **Seerat Shirazi**, Department of Neurosurgery, Leeds Teaching Hospitals, Leeds; **Catherine E Gilkes**, Department of Neurosurgery, The Walton Centre, Liverpool; **Christopher P Millward**, Department of Neurosurgery, The Walton Centre, Liverpool; **Ahmad MS Ali**, Department of Neurosurgery, The Walton Centre, Liverpool; **Dimitris Paraskevopoulos**, Department of Neurosurgery, Barts and The Royal London Hospital, London; **Jarnail Bal**, Department of Neurosurgery, Barts and The Royal London Hospital, London; **Samir Matloob**, Department of Neurosurgery, Barts and The Royal London Hospital, London; **Rhannon Lobo**, Department of Neurosurgery, Barts and The Royal London Hospital, London; **Nigel Mendoza**, Department of Neurosurgery, Charing Cross Hospital, London; **Ramesh Nair**, Department of Neurosurgery, Charing Cross Hospital, London; **Arthur Dalton**, Department of Neurosurgery, Charing Cross Hospital, London; **Adarsh Nadig**, Department of Neurosurgery, Charing Cross Hospital, London; **Lucas Hernandez**, Department of Neurosurgery, Charing Cross Hospital, London; **Nick Thomas**, Department of Neurosurgery, King's College Hospital, London; **Eleni Maratos**, Department of Neurosurgery, King's College Hospital, London; **Jonathan Shapey**, Department of Neurosurgery, King's College Hospital, London; **Sinan Al-Barazi**, Department of Neurosurgery, King's College Hospital, London; **Asfand Baig Mirza**, Department of Neurosurgery, King's College Hospital, London; **Mohamed Okasha**, Department of Neurosurgery, King's College Hospital, London; **Prabhjot Singh Malhotra**, Department of Neurosurgery, King's College Hospital, London; **Razna Ahmed**, Department of Neurosurgery, King's College Hospital, London; **Neil L Dorward**, Department of Neurosurgery, National Hospital for Neurology and Neurosurgery, London; **Joan Grieve**, Department of Neurosurgery, National Hospital for Neurology and Neurosurgery, London; **Hani J Marcus**, Department of Neurosurgery, National Hospital for Neurology and Neurosurgery, London; **Parag Sayal**, Department of Neurosurgery, National Hospital for Neurology and Neurosurgery, London; **David Choi**, Department of Neurosurgery, National Hospital for Neurology and Neurosurgery, London; **Ivan Cabrilo**, Department of Neurosurgery, National Hospital for Neurology and Neurosurgery, London; **Hugo Layard Horsfall**, Department of Neurosurgery, National Hospital for Neurology and Neurosurgery, London; **Jonathan Pollock**, Department of Neurosurgery, Barking, Havering & Redbridge University Hospitals, London; **Alireza Shoakazemi**, Department of Neurosurgery, Barking, Havering & Redbridge University Hospitals, London; **Oscar Maccormac**, Department of Neurosurgery, Barking, Havering & Redbridge University Hospitals, London; **Guru N K Amirthalingam**, Department of Neurosurgery, Barking, Havering & Redbridge University Hospitals, London; **Andrew Martin**, Department of Neurosurgery, St George’s University Hospitals Trust, London; **Simon Stapleton**, Department of Neurosurgery, St George’s University Hospitals Trust, London; **Florence Hogg**, Department of Neurosurgery, St George’s University Hospitals Trust, London; **Daniel Richardson**, Department of Neurosurgery, St George’s University Hospitals Trust, London; **Kanna Gnanalingham**, Department of Neurosurgery, Salford Royal Trust, Manchester; **Omar Pathmanaban**, Department of Neurosurgery, Salford Royal Trust, Manchester; **Daniel M Fountain**, Department of Neurosurgery, Salford Royal Trust, Manchester; **Raj Bhalla**, Department of Otorhinolaryngology, Salford Royal Trust, Manchester; **Cathal J Hannan**, Department of Neurosurgery, Salford Royal Trust, Manchester; **Annabel Chadwick**, Department of Neurosurgery, Salford Royal Trust, Manchester; **Alistair Jenkins**, Department of Neurosurgery, Royal Victoria Infirmary, Newcastle; **Claire Nicholson**, Department of Neurosurgery, Royal Victoria Infirmary, Newcastle; **Syed Shumon**, Department of Neurosurgery, Royal Victoria Infirmary, Newcastle; **Mohamed Youssef**, Department of Neurosurgery, Royal Victoria Infirmary, Newcastle; **Callum Allison**, Department of Neurosurgery, Royal Victoria Infirmary, Newcastle; **Graham Dow**, Department of Neurosurgery, Queen's Medical Centre Nottingham, Nottingham; **Iain Robertson**, Department of Neurosurgery, Queen's Medical Centre Nottingham, Nottingham; **Laurence Johann Glancz**, Department of Neurosurgery, Queen's Medical Centre Nottingham, Nottingham; **Murugan Sitaraman**, Department of Neurosurgery, Queen's Medical Centre Nottingham, Nottingham; **Ashwin Kumaria**, Department of Neurosurgery, Queen's Medical Centre Nottingham, Nottingham; **Ananyo Bagchi**, Department of Neurosurgery, Queen's Medical Centre Nottingham, Nottingham; **Simon Cudlip**, Department of Neurosurgery, John Radcliffe Hospital, Oxford University Hospitals, Oxford; **Jane Halliday**, Department of Neurosurgery, John Radcliffe Hospital, Oxford University Hospitals, Oxford; **Rory J Piper**, Department of Neurosurgery, John Radcliffe Hospital, Oxford University Hospitals, Oxford; **Alexandros Boukas**, Department of Neurosurgery, John Radcliffe Hospital, Oxford University Hospitals, Oxford; **Meriem Amarouche**, Department of Neurosurgery, John Radcliffe Hospital, Oxford University Hospitals, Oxford; **Damjan Veljanoski**, Department of Neurosurgery, John Radcliffe Hospital, Oxford University Hospitals, Oxford; **Samiul Muquit**, Department of Neurosurgery, University Hospitals Plymouth, Plymouth; **Ellie Edlmann**, Department of Neurosurgery, University Hospitals Plymouth, Plymouth; **Haritha Maripi**, Department of Neurosurgery, University Hospitals Plymouth, Plymouth; **Yi Wang**, Department of Neurosurgery, University Hospitals Plymouth, Plymouth; **Mehnaz Hossain**, Department of Neurosurgery, University Hospitals Plymouth, Plymouth; **Andrew Alalade**, Department of Neurosurgery, Lancashire Teaching Hospitals NHS Foundation Trust, Preston; **Syed Maroof**, Department of Neurosurgery, Lancashire Teaching Hospitals NHS Foundation Trust, Preston; **Pradnya Patkar**, Department of Neurosurgery, Lancashire Teaching Hospitals NHS Foundation Trust, Preston; **Saurabh Sinha**, Department of Neurosurgery, Royal Hallamshire Hospital & Sheffield Children’s Hospital, Sheffield; **Showkat Mirza**, Department of Otorhinolaryngology, Royal Hallamshire Hospital & Sheffield Children’s Hospital, Sheffield; **Duncan Henderson**, Department of Neurosurgery, Royal Hallamshire Hospital & Sheffield Children’s Hospital, Sheffield; **Mohammad Saud Khan**, Department of Neurosurgery, Royal Hallamshire Hospital & Sheffield Children’s Hospital, Sheffield; **Nijaguna Mathad**, Department of Neurosurgery, University Hospital Southampton, Southampton; **Jonathan Hempenstall**, Department of Neurosurgery, University Hospital Southampton, Southampton; **Difei Wang**, Department of Neurosurgery, University Hospital Southampton, Southampton; **Pavan Marwaha**, Department of Neurosurgery, University Hospital Southampton, Southampton; **Simon Shaw**, Department of Neurosurgery, Royal Stoke University Hospital, Stoke; **Georgios Solomou**, Department of Neurosurgery, Royal Stoke University Hospital, Stoke; **Alina Shrestha**, Department of Neurosurgery, Royal Stoke University Hospital, Stoke. †Joint first authorship, ‡Joint senior authorship.

## Author contributions

This is a group authorship model paper where all authors contributed to data collection and approved the final manuscript. All authors listed have made a substantial, direct, and intellectual contribution to the work and approved it for publication. Study design and analysis were performed by AD and DZK. The first draft of the manuscript was written by AD and DZK. Revision of manuscript and supervision was provided by HJM and DS.
