# Supplementary material for: Machine learning driven prediction of cerebrospinal fluid rhinorrhoea following endonasal skull base surgery: A multicentre prospective observational study
Source: Front Oncol. 2023 Mar 23;13:1046519. doi: 10.3389/fonc.2023.1046519 (PMC10076706; doi:10.3389/fonc.2023.1046519)
Supplement: Supplementary file 1 [file DataSheet_1.pdf]

Supplementary Materials:

Supplementary material 1: List of authors and collaborators. *indicates joint first authorship. †indicates joint senior authorship.

1a. Authors

**Team**	**Name**
Wellcome/EPSRC Centre for Interventional and Surgical Sciences, University College London, London	Adrito Das*
Department of Neurosurgery, National Hospital for Neurology and Neurosurgery, London	Danyal Z Khan*
Wellcome/EPSRC Centre for Interventional and Surgical Sciences, University College London, London	Danail Stoyanov†
Department of Neurosurgery, National Hospital for Neurology and Neurosurgery, London	Hani J Marcus†
Oxford University Global Surgery Group, Nuffield Department of Surgical Sciences, University of Oxford, Oxford	Soham Bandyopadhyay
Department of Neurology, University Hospital of Wales, Cardiff University, Cardiff	Benjamin E Schroeder
Division of Neurosurgery, Cambridge University Hospitals Trust, Cambridge	Vikesh Patel
Birmingham Medical School, University of Birmingham, Birmingham	Alice O'Donnell
NANSIG	Neurology and Neurosurgery Interest Group
BNTRC	British Neurosurgical Trainee Research Collaborative
Department of Neurosurgery, Aberdeen Royal Infirmary, Aberdeen	Anastasios Giamouriadis
Department of Neurosurgery, Aberdeen Royal Infirmary, Aberdeen	Pragnesh Bhatt
Department of Otorhinolaryngology, Aberdeen Royal Infirmary, Aberdeen	Bhaskar Ram
Department of Neurosurgery, Aberdeen Royal Infirmary, Aberdeen	Adithya Varma
Department of Neurosurgery, Aberdeen Royal Infirmary, Aberdeen	Ioannis Georgiou
Department of Neurosurgery, Royal Victoria Hospital, Belfast	Philip Weir
Department of Otorhinolaryngology, Royal Victoria Hospital, Belfast	Brendan Hanna
Department of Neurosurgery, Royal Victoria Hospital, Belfast	Theodore C Hirst
Department of Neurosurgery, Royal Victoria Hospital, Belfast	Patrick McAleavey
Department of Neurosurgery, Queen Elizabeth Hospital Birmingham, Birmingham	Alessandro Paluzzi
Department of Neurosurgery, Queen Elizabeth Hospital Birmingham, Birmingham	Georgios Tsermoulas
Department of Otorhinolaryngology, Queen Elizabeth Hospital Birmingham, Birmingham	Shahzada Ahmed
Department of Neurosurgery, Queen Elizabeth Hospital Birmingham, Birmingham	Wai Cheong Soon
Department of Neurosurgery, Queen Elizabeth Hospital Birmingham, Birmingham	Yasir Arafat Chowdhury
Department of Neurosurgery, Queen Elizabeth Hospital Birmingham, Birmingham	Suhaib Abualsaud
Department of Neurosurgery, Queen Elizabeth Hospital Birmingham, Birmingham	Shumail Mahmood
Department of Otorhinolaryngology, Queen Elizabeth Hospital Birmingham, Birmingham	Paresh Naik
Department of Neurosurgery, Queen Elizabeth Hospital Birmingham, Birmingham	Zohra Haiderkhan
Department of Neurosurgery, Hurstwood Park Neurosciences Centre and Royal Sussex County Hospital, Brighton	Rafid Al-Mahfoudh
Department of Neurosurgery, Hurstwood Park Neurosciences Centre and Royal Sussex County Hospital, Brighton	Andrea Perera
Department of Neurosurgery, Hurstwood Park Neurosciences Centre and Royal Sussex County Hospital, Brighton	Mircea Rus
Department of Neurosurgery, Southmead Hospital Bristol, Bristol	Adam Williams
Department of Neurosurgery, Southmead Hospital Bristol, Bristol	Charles Hand
Department of Neurosurgery, Southmead Hospital Bristol, Bristol	Kumar Abhinav
Department of Neurosurgery, Southmead Hospital Bristol, Bristol	Cristina Cernei
Department of Neurosurgery, Southmead Hospital Bristol, Bristol	Aiman Dilnawaz
Division of Neurosurgery, Cambridge University Hospitals Trust, Cambridge	Richard Mannion
Division of Neurosurgery, Cambridge University Hospitals Trust, Cambridge	Thomas Santarius
Division of Otorhinolaryngology, Cambridge University Hospitals Trust, Cambridge	James Tysome
Division of Otorhinolaryngology, Cambridge University Hospitals Trust, Cambridge	Rishi Sharma
Division of Neurosurgery, Cambridge University Hospitals Trust, Cambridge	Angelos G Kolias
Division of Otorhinolaryngology, Cambridge University Hospitals Trust, Cambridge	Neil Donnelly
Division of Neurosurgery, Cambridge University Hospitals Trust, Cambridge	Vikesh Patel
Division of Neurosurgery, Cambridge University Hospitals Trust, Cambridge	Ashwin Venkatesh

Department of Neurosurgery, University Hospital of Wales, Cardiff	Caroline Hayhurst
Department of Neurosurgery, University Hospital of Wales, Cardiff	Amr Mohamed
Department of Otorhinolaryngology, University Hospital of Wales, Cardiff	Benjamin Stew
Department of Neurosurgery, University Hospital of Wales, Cardiff	Joseph Merola
Department of Neurosurgery, University Hospital of Wales, Cardiff	Setthasorn Zhi Yang, Ooi
Department of Neurosurgery, Cork University Hospitals, Ireland	Mahmoud Kamel
Department of Otorhinolaryngology, Cork University Hospitals, Ireland	Mohammad Habibullah Khan
Department of Neurosurgery, Cork University Hospitals, Ireland	Sahibzada Abrar
Department of Neurosurgery, Cork University Hospitals, Ireland	Christopher Mckeon
Department of Neurosurgery, Cork University Hospitals, Ireland	Dan McSweeney
Department of Neurosurgery, National Neurosurgical Centre, Beaumont Hospital, Ireland	Mohsen Javadpour
Department of Otorhinolaryngology, National Neurosurgical Centre, Beaumont Hospital, Ireland	Peter Lacy
Department of Neurosurgery, National Neurosurgical Centre, Beaumont Hospital, Ireland	Daniel Murray
Department of Neurosurgery, National Neurosurgical Centre, Beaumont Hospital, Ireland	Elena Roman
Department of Neurosurgery, Ninewells Hospital, Dundee	Kismet Hossain-Ibrahim
Department of Otorhinolaryngology, Ninewells Hospital, Dundee	Peter Ross
Department of Neurosurgery, Ninewells Hospital, Dundee	David Bennett
Department of Neurosurgery, Ninewells Hospital, Dundee	Nathan McSorley
Department of Neurosurgery, Ninewells Hospital, Dundee	Adam Hounat
Department of Clinical Neurosciences, BioQuarter, Edinburgh	Patrick Statham
Department of Clinical Neurosciences, BioQuarter, Edinburgh	Mark Hughes
Department of Clinical Neurosciences, BioQuarter, Edinburgh	Alhafidz Hamdan
Department of Clinical Neurosciences, BioQuarter, Edinburgh	Caroline Scott
Department of Clinical Neurosciences, BioQuarter, Edinburgh	Jisinga Joshi
Department of Neurosurgery, Hull University Teaching Hospitals, Hull	Anuj Bahl
Department of Neurosurgery, Hull University Teaching Hospitals, Hull	Anna Bjornson
Department of Neurosurgery, Hull University Teaching Hospitals, Hull	Daniel Gatt
Department of Neurosurgery, Leeds Teaching Hospitals, Leeds	Nick Phillips
Department of Neurosurgery, Leeds Teaching Hospitals, Leeds	Neeraj Kalra
Department of Neurosurgery, Leeds Teaching Hospitals, Leeds	Melissa Bautista
Department of Neurosurgery, Leeds Teaching Hospitals, Leeds	Seerat Shirazi
Department of Neurosurgery, The Walton Centre, Liverpool	Catherine E Gilkes
Department of Neurosurgery, The Walton Centre, Liverpool	Christopher P Millward
Department of Neurosurgery, The Walton Centre, Liverpool	Ahmad MS Ali
Department of Neurosurgery, Barts and The Royal London Hospital, London	Dimitris Paraskevopoulos
Department of Neurosurgery, Barts and The Royal London Hospital, London	Jarnail Bal
Department of Neurosurgery, Barts and The Royal London Hospital, London	Samir Matloob
Department of Neurosurgery, Barts and The Royal London Hospital, London	Rhannon Lobo
Department of Neurosurgery, Charing Cross Hospital, London	Nigel Mendoza
Department of Neurosurgery, Charing Cross Hospital, London	Ramesh Nair
Department of Neurosurgery, Charing Cross Hospital, London	Arthur Dalton
Department of Neurosurgery, Charing Cross Hospital, London	Adarsh Nadig
Department of Neurosurgery, Charing Cross Hospital, London	Lucas Hernandez
Department of Neurosurgery, King's College Hospital, London	Nick Thomas
Department of Neurosurgery, King's College Hospital, London	Eleni Maratos
Department of Neurosurgery, King's College Hospital, London	Jonathan Shapey
Department of Neurosurgery, King's College Hospital, London	Sinan Al-Barazi
Department of Neurosurgery, King's College Hospital, London	Asfand Baig Mirza
Department of Neurosurgery, King's College Hospital, London	Mohamed Okasha
Department of Neurosurgery, King's College Hospital, London	Prabhjot Singh Malhotra
Department of Neurosurgery, King's College Hospital, London	Razna Ahmed

Department of Neurosurgery, National Hospital for Neurology and Neurosurgery, London	Neil L Dorward
Department of Neurosurgery, National Hospital for Neurology and Neurosurgery, London	Joan Grieve
Department of Neurosurgery, National Hospital for Neurology and Neurosurgery, London	Hani J Marcus
Department of Neurosurgery, National Hospital for Neurology and Neurosurgery, London	Parag Sayal
Department of Neurosurgery, National Hospital for Neurology and Neurosurgery, London	David Choi
Department of Neurosurgery, National Hospital for Neurology and Neurosurgery, London	Ivan Cabrilo
Department of Neurosurgery, National Hospital for Neurology and Neurosurgery, London	Hugo Layard Horsfall
Department of Neurosurgery, Barking, Havering & Redbridge University Hospitals, London	Jonathan Pollock
Department of Neurosurgery, Barking, Havering & Redbridge University Hospitals, London	Alireza Shoakazemi
Department of Neurosurgery, Barking, Havering & Redbridge University Hospitals, London	Oscar Maccormac
Department of Neurosurgery, Barking, Havering & Redbridge University Hospitals, London	Guru N K Amirthalingam
Department of Neurosurgery, St George's University Hospitals Trust, London	Andrew Martin
Department of Neurosurgery, St George's University Hospitals Trust, London	Simon Stapleton
Department of Neurosurgery, St George's University Hospitals Trust, London	Florence Hogg
Department of Neurosurgery, St George's University Hospitals Trust, London	Daniel Richardson
Department of Neurosurgery, Salford Royal Trust, Manchester	Kanna Gnanalingham
Department of Neurosurgery, Salford Royal Trust, Manchester	Omar Pathmanaban
Department of Neurosurgery, Salford Royal Trust, Manchester	Daniel M Fountain
Department of Otorhinolaryngology, Salford Royal Trust, Manchester	Raj Bhalla
Department of Neurosurgery, Salford Royal Trust, Manchester	Cathal J Hannan
Department of Neurosurgery, Salford Royal Trust, Manchester	Annabel Chadwick
Department of Neurosurgery, Royal Victoria Infirmary, Newcastle	Alistair Jenkins
Department of Neurosurgery, Royal Victoria Infirmary, Newcastle	Claire Nicholson
Department of Neurosurgery, Royal Victoria Infirmary, Newcastle	Syed Shumon
Department of Neurosurgery, Royal Victoria Infirmary, Newcastle	Mohamed Youssef
Department of Neurosurgery, Royal Victoria Infirmary, Newcastle	Callum Allison
Department of Neurosurgery, Queen's Medical Centre Nottingham, Nottingham	Graham Dow
Department of Neurosurgery, Queen's Medical Centre Nottingham, Nottingham	Iain Robertson
Department of Neurosurgery, Queen's Medical Centre Nottingham, Nottingham	Laurence Glancz
Department of Neurosurgery, Queen's Medical Centre Nottingham, Nottingham	Murugan Sitaraman
Department of Neurosurgery, Queen's Medical Centre Nottingham, Nottingham	Ashwin Kumaria
Department of Neurosurgery, Queen's Medical Centre Nottingham, Nottingham	Ananyo Bagchi
Department of Neurosurgery, John Radcliffe Hospital, Oxford University Hospitals, Oxford	Simon Cudlip
Department of Neurosurgery, John Radcliffe Hospital, Oxford University Hospitals, Oxford	Jane Halliday
Department of Neurosurgery, John Radcliffe Hospital, Oxford University Hospitals, Oxford	Rory J Piper
Department of Neurosurgery, John Radcliffe Hospital, Oxford University Hospitals, Oxford	Alexandros Boukas
Department of Neurosurgery, John Radcliffe Hospital, Oxford University Hospitals, Oxford	Meriem Amarouche
Department of Neurosurgery, John Radcliffe Hospital, Oxford University Hospitals, Oxford	Damjan Veljanoski
Department of Neurosurgery, University Hospitals Plymouth, Plymouth	Sam Muquit
Department of Neurosurgery, University Hospitals Plymouth, Plymouth	Ellie Edlmann
Department of Neurosurgery, University Hospitals Plymouth, Plymouth	Haritha Maripi
Department of Neurosurgery, University Hospitals Plymouth, Plymouth	Yi Wang
Department of Neurosurgery, University Hospitals Plymouth, Plymouth	Mehnaz Hossain
Department of Neurosurgery, Lancashire Teaching Hospitals NHS Foundation Trust, Preston	Andrew Alalade
Department of Neurosurgery, Lancashire Teaching Hospitals NHS Foundation Trust, Preston	Syed Maroof
Department of Neurosurgery, Lancashire Teaching Hospitals NHS Foundation Trust, Preston	Pradnya Patkar
Department of Neurosurgery, Royal Hallamshire Hospital & Sheffield Children's Hospital, Sheffield	Saurabh Sinha
Department of Otorhinolaryngology, Royal Hallamshire Hospital & Sheffield Children's Hospital, Sheffield	Showkat Mirza
Department of Neurosurgery, Royal Hallamshire Hospital & Sheffield Children's Hospital, Sheffield	Duncan Henderson
Department of Neurosurgery, Royal Hallamshire Hospital & Sheffield Children's Hospital, Sheffield	Mohammad Saud Khan
Department of Neurosurgery, University Hospital Southampton, Southampton	Nijaguna Mathad

Department of Neurosurgery, University Hospital Southampton, Southampton	Jonathan Hempenstall
Department of Neurosurgery, University Hospital Southampton, Southampton	Difei Wang
Department of Neurosurgery, University Hospital Southampton, Southampton	Pavan Marwaha
Department of Neurosurgery, Royal Stoke University Hospital, Stoke	Simon Shaw
Department of Neurosurgery, Royal Stoke University Hospital, Stoke	Georgios Solomou
Department of Neurosurgery, Royal Stoke University Hospital, Stoke	Alina Shrestha

1b. Collaborators (data validators)

**Team**	**Name**
Department of Neurosurgery, Aberdeen Royal Infirmary, Aberdeen	Andrew Fraser
Department of Neurosurgery, Royal Victoria Hospital, Belfast	Theodore Hirst
Department of Neurosurgery, Queen Elizabeth Hospital Birmingham, Birmingham	Yasir Chowdhury
Department of Neurosurgery, Hurstwood Park Neurosciences Centre and Royal Sussex County Hospital, Brighton	Sobiya Bilal
Department of Neurosurgery, Southmead Hospital Bristol, Bristol	Jack Wildman
Division of Neurosurgery, Cambridge University Hospitals Trust, Cambridge	Ashwin Venkatesh
Department of Neurosurgery, University Hospital of Wales, Cardiff	Priya Babu
Department of Neurosurgery, Cork University Hospitals, Ireland	Cian Carey
Department of Neurosurgery, National Neurosurgical Centre, Beaumont Hospital, Ireland	Renitha Reddi Bathuni
Department of Neurosurgery, Ninewells Hospital, Dundee	Kismet Hossain-Ibrahim
Department of Neurosurgery, The Western General Hospital, Edinburgh	Joseph Nathaniel Brennan
Department of Neurosurgery, Hull University Teaching Hospitals, Hull	Anna Bjornson
Department of Neurosurgery, Leeds Teaching Hospitals, Leeds	Howra Ktayen
Department of Neurosurgery, The Walton Centre, Liverpool	Sandhya T Trichinopoly
Department of Neurosurgery, Barts and The Royal London Hospital, London	Samir Matloob
Department of Neurosurgery, Charing Cross Hospital, London	Adarsh Nadig
Department of Neurosurgery, King's College Hospital, London	Mohamed Okasha
Department of Neurosurgery, National Hospital for Neurology and Neurosurgery, London	Danyal Khan
Department of Neurosurgery, Barking, Havering & Redbridge University Hospitals, London	Alireza Shoakazemi
Department of Neurosurgery, St George’s University Hospitals Trust, London	Florence Hogg
Department of Neurosurgery, Salford Royal Trust, Manchester	Seun Sobawale
Department of Neurosurgery, Royal Victoria Infirmary, Newcastle	Amir Suliman
Department of Neurosurgery, Queen's Medical Centre Nottingham, Nottingham	Ashwin Kumaria
Department of Neurosurgery, John Radcliffe Hospital, Oxford University Hospitals, Oxford	Rory Piper
Department of Neurosurgery, John Radcliffe Hospital, Oxford University Hospitals, Oxford	Will Owen
Department of Neurosurgery, University Hospitals Plymouth, Plymouth	Ellie Edlmann
Department of Neurosurgery, Lancashire Teaching Hospitals NHS Foundation Trust, Preston	Afaq Sartaj
Department of Neurosurgery, Royal Hallamshire Hospital & Sheffield Children’s Hospital, Sheffield	Edward Goacher
Department of Neurosurgery, University Hospital Southampton, Southampton	Euan Strachan
Department of Neurosurgery, Royal Stoke University Hospital, Stoke	Giorgios Solomou
