# Supplementary material for: Machine learning driven prediction of cerebrospinal fluid rhinorrhoea following endonasal skull base surgery: A multicentre prospective observational study
Source: Front Oncol. 2023 Mar 23;13:1046519. doi: 10.3389/fonc.2023.1046519 (PMC10076706; doi:10.3389/fonc.2023.1046519)
Supplement: Supplementary file 2 [file DataSheet_2.docx]

Supplementary Material

**Table 1:** List of CRANIAL consortium authors.
*indicates joint first authorship. ^†^indicates joint senior authorship.

| **Team** | **Name** |
| --- | --- |
| Wellcome/EPSRC Centre for Interventional and Surgical Sciences, University College London, London Department of Neurosurgery, National Hospital for Neurology and Neurosurgery, London  Wellcome/EPSRC Centre for Interventional and Surgical Sciences, University College London, London | Adrito Das*  Danyal Z Khan*  Danail Stoyanov^†^ |
| Department of Neurosurgery, National Hospital for Neurology and Neurosurgery, London | Hani J Marcus^†^ |
| Oxford University Global Surgery Group, Nuffield Department of Surgical Sciences, University of Oxford, Oxford | Soham Bandyopadhyay |
| Department of Neurology, University Hospital of Wales, Cardiff University, Cardiff | Benjamin E Schroeder |
| Division of Neurosurgery, Cambridge University Hospitals Trust, Cambridge | Vikesh Patel |
| Birmingham Medical School, University of Birmingham, Birmingham | Alice O’Donnell |
| NANSIG | Neurology and Neurosurgery Interest Group |
| BNTRC | British Neurosurgical Trainee Research Collaborative |
| Department of Neurosurgery, Aberdeen Royal Infirmary, Aberdeen | Anastasios Giamouriadis |
| Department of Neurosurgery, Aberdeen Royal Infirmary, Aberdeen | Pragnesh Bhatt |
| Department of Otorhinolaryngology, Aberdeen Royal Infirmary, Aberdeen | Bhaskar Ram |
| Department of Neurosurgery, Aberdeen Royal Infirmary, Aberdeen | Adithya Varma |
| Department of Neurosurgery, Aberdeen Royal Infirmary, Aberdeen | Ioannis Georgiou |
| Department of Neurosurgery, Royal Victoria Hospital, Belfast | Philip Weir |
| Department of Otorhinolaryngology, Royal Victoria Hospital, Belfast | Brendan Hanna |
| Department of Neurosurgery, Royal Victoria Hospital, Belfast | Theodore C Hirst |
| Department of Neurosurgery, Royal Victoria Hospital, Belfast | Patrick McAleavey |
| Department of Neurosurgery, Queen Elizabeth Hospital Birmingham, Birmingham | Alessandro Paluzzi |
| Department of Neurosurgery, Queen Elizabeth Hospital Birmingham, Birmingham | Georgios Tsermoulas |
| Department of Otorhinolaryngology, Queen Elizabeth Hospital Birmingham, Birmingham | Shahzada Ahmed |
| Department of Neurosurgery, Queen Elizabeth Hospital Birmingham, Birmingham | Wai Cheong Soon |
| Department of Neurosurgery, Queen Elizabeth Hospital Birmingham, Birmingham | Yasir Arafat Chowdhury |
| Department of Neurosurgery, Queen Elizabeth Hospital Birmingham, Birmingham | Suhaib Abualsaud |
| Department of Neurosurgery, Queen Elizabeth Hospital Birmingham, Birmingham | Shumail Mahmood |
| Department of Otorhinolaryngology, Queen Elizabeth Hospital Birmingham, Birmingham | Paresh Naik |
| Department of Neurosurgery, Queen Elizabeth Hospital Birmingham, Birmingham | Zohra Haiderkhan |
| Department of Neurosurgery, Hurstwood Park Neurosciences Centre and Royal Sussex County Hospital, Brighton | Rafid Al-Mahfoudh |
| Department of Neurosurgery, Hurstwood Park Neurosciences Centre and Royal Sussex County Hospital, Brighton | Andrea Perera |
| Department of Neurosurgery, Hurstwood Park Neurosciences Centre and Royal Sussex County Hospital, Brighton | Mircea Rus |
| Department of Neurosurgery, Southmead Hospital Bristol, Bristol | Adam Williams |
| Department of Neurosurgery, Southmead Hospital Bristol, Bristol | Charles Hand |
| Department of Neurosurgery, Southmead Hospital Bristol, Bristol | Kumar Abhinav |
| Department of Neurosurgery, Southmead Hospital Bristol, Bristol | Cristina Cernei |
| Department of Neurosurgery, Southmead Hospital Bristol, Bristol | Aiman Dilnawaz |
| Division of Neurosurgery, Cambridge University Hospitals Trust, Cambridge | Richard Mannion |
| Division of Neurosurgery, Cambridge University Hospitals Trust, Cambridge | Thomas Santarius |
| Division of Otorhinolaryngology, Cambridge University Hospitals Trust, Cambridge | James Tysome |
| Division of Otorhinolaryngology, Cambridge University Hospitals Trust, Cambridge | Rishi Sharma |
| Division of Neurosurgery, Cambridge University Hospitals Trust, Cambridge | Angelos G Kolias |
| Division of Otorhinolaryngology, Cambridge University Hospitals Trust, Cambridge | Neil Donnelly |
| Division of Neurosurgery, Cambridge University Hospitals Trust, Cambridge | Vikesh Patel |
| Division of Neurosurgery, Cambridge University Hospitals Trust, Cambridge | Ashwin Venkatesh |
| Department of Neurosurgery, University Hospital of Wales, Cardiff | Caroline Hayhurst |
| Department of Neurosurgery, University Hospital of Wales, Cardiff | Amr Mohamed |
| Department of Otorhinolaryngology, University Hospital of Wales, Cardiff | Benjamin Stew |
| Department of Neurosurgery, University Hospital of Wales, Cardiff | Joseph Merola |
| Department of Neurosurgery, University Hospital of Wales, Cardiff | Setthasorn Zhi Yang, Ooi |
| Department of Neurosurgery, Cork University Hospitals, Ireland | Mahmoud Kamel |
| Department of Otorhinolaryngology, Cork University Hospitals, Ireland | Mohammad Habibullah Khan |
| Department of Neurosurgery, Cork University Hospitals, Ireland | Sahibzada Abrar |
| Department of Neurosurgery, Cork University Hospitals, Ireland | Christopher Mckeon |
| Department of Neurosurgery, Cork University Hospitals, Ireland | Dan McSweeney |
| Department of Neurosurgery, National Neurosurgical Centre, Beaumont Hospital, Ireland | Mohsen Javadpour |
| Department of Otorhinolaryngology, National Neurosurgical Centre, Beaumont Hospital, Ireland | Peter Lacy |
| Department of Neurosurgery, National Neurosurgical Centre, Beaumont Hospital, Ireland | Daniel Murray |
| Department of Neurosurgery, National Neurosurgical Centre, Beaumont Hospital, Ireland | Elena Roman |
| Department of Neurosurgery, Ninewells Hospital, Dundee | Kismet Hossain-Ibrahim |
| Department of Otorhinolaryngology, Ninewells Hospital, Dundee | Peter Ross |
| Department of Neurosurgery, Ninewells Hospital, Dundee | David Bennett |
| Department of Neurosurgery, Ninewells Hospital, Dundee | Nathan McSorley |
| Department of Neurosurgery, Ninewells Hospital, Dundee | Adam Hounat |
| Department of Clinical Neurosciences, BioQuarter, Edinburgh | Patrick Statham |
| Department of Clinical Neurosciences, BioQuarter, Edinburgh | Mark Hughes |
| Department of Clinical Neurosciences, BioQuarter, Edinburgh | Alhafidz Hamdan |
| Department of Clinical Neurosciences, BioQuarter, Edinburgh | Caroline Scott |
| Department of Clinical Neurosciences, BioQuarter, Edinburgh | Jisinga Joshi |
| Department of Neurosurgery, Hull University Teaching Hospitals, Hull | Anuj Bahl |
| Department of Neurosurgery, Hull University Teaching Hospitals, Hull | Anna Bjornson |
| Department of Neurosurgery, Hull University Teaching Hospitals, Hull | Daniel Gatt |
| Department of Neurosurgery, Leeds Teaching Hospitals, Leeds | Nick Phillips |
| Department of Neurosurgery, Leeds Teaching Hospitals, Leeds | Neeraj Kalra |
| Department of Neurosurgery, Leeds Teaching Hospitals, Leeds | Melissa Bautista |
| Department of Neurosurgery, Leeds Teaching Hospitals, Leeds | Seerat Shirazi |
| Department of Neurosurgery, The Walton Centre, Liverpool | Catherine E Gilkes |
| Department of Neurosurgery, The Walton Centre, Liverpool | Christopher P Millward |
| Department of Neurosurgery, The Walton Centre, Liverpool | Ahmad MS Ali |
| Department of Neurosurgery, Barts and The Royal London Hospital, London | Dimitris Paraskevopoulos |
| Department of Neurosurgery, Barts and The Royal London Hospital, London | Jarnail Bal |
| Department of Neurosurgery, Barts and The Royal London Hospital, London | Samir Matloob |
| Department of Neurosurgery, Barts and The Royal London Hospital, London | Rhannon Lobo |
| Department of Neurosurgery, Charing Cross Hospital, London | Nigel Mendoza |
| Department of Neurosurgery, Charing Cross Hospital, London | Ramesh Nair |
| Department of Neurosurgery, Charing Cross Hospital, London | Arthur Dalton |
| Department of Neurosurgery, Charing Cross Hospital, London | Adarsh Nadig |
| Department of Neurosurgery, Charing Cross Hospital, London | Lucas Hernandez |
| Department of Neurosurgery, King's College Hospital, London | Nick Thomas |
| Department of Neurosurgery, King's College Hospital, London | Eleni Maratos |
| Department of Neurosurgery, King's College Hospital, London | Jonathan Shapey |
| Department of Neurosurgery, King's College Hospital, London | Sinan Al-Barazi |
| Department of Neurosurgery, King's College Hospital, London | Asfand Baig Mirza |
| Department of Neurosurgery, King's College Hospital, London | Mohamed Okasha |
| Department of Neurosurgery, King's College Hospital, London | Prabhjot Singh Malhotra |
| Department of Neurosurgery, King's College Hospital, London | Razna Ahmed |
| Department of Neurosurgery, National Hospital for Neurology and Neurosurgery, London | Neil L Dorward |
| Department of Neurosurgery, National Hospital for Neurology and Neurosurgery, London | Joan Grieve |
| Department of Neurosurgery, National Hospital for Neurology and Neurosurgery, London | Hani J Marcus |
| Department of Neurosurgery, National Hospital for Neurology and Neurosurgery, London | Parag Sayal |
| Department of Neurosurgery, National Hospital for Neurology and Neurosurgery, London | David Choi |
| Department of Neurosurgery, National Hospital for Neurology and Neurosurgery, London | Ivan Cabrilo |
| Department of Neurosurgery, National Hospital for Neurology and Neurosurgery, London | Hugo Layard Horsfall |
| Department of Neurosurgery, Barking, Havering & Redbridge University Hospitals, London | Jonathan Pollock |
| Department of Neurosurgery, Barking, Havering & Redbridge University Hospitals, London | Alireza Shoakazemi |
| Department of Neurosurgery, Barking, Havering & Redbridge University Hospitals, London | Oscar Maccormac |
| Department of Neurosurgery, Barking, Havering & Redbridge University Hospitals, London | Guru N K Amirthalingam |
| Department of Neurosurgery, St George’s University Hospitals Trust, London | Andrew Martin |
| Department of Neurosurgery, St George’s University Hospitals Trust, London | Simon Stapleton |
| Department of Neurosurgery, St George’s University Hospitals Trust, London | Florence Hogg |
| Department of Neurosurgery, St George’s University Hospitals Trust, London | Daniel Richardson |
| Department of Neurosurgery, Salford Royal Trust, Manchester | Kanna Gnanalingham |
| Department of Neurosurgery, Salford Royal Trust, Manchester | Omar Pathmanaban |
| Department of Neurosurgery, Salford Royal Trust, Manchester | Daniel M Fountain |
| Department of Otorhinolaryngology, Salford Royal Trust, Manchester | Raj Bhalla |
| Department of Neurosurgery, Salford Royal Trust, Manchester | Cathal J Hannan |
| Department of Neurosurgery, Salford Royal Trust, Manchester | Annabel Chadwick |
| Department of Neurosurgery, Royal Victoria Infirmary, Newcastle | Alistair Jenkins |
| Department of Neurosurgery, Royal Victoria Infirmary, Newcastle | Claire Nicholson |
| Department of Neurosurgery, Royal Victoria Infirmary, Newcastle | Syed Shumon |
| Department of Neurosurgery, Royal Victoria Infirmary, Newcastle | Mohamed Youssef |
| Department of Neurosurgery, Royal Victoria Infirmary, Newcastle | Callum Allison |
| Department of Neurosurgery, Queen's Medical Centre Nottingham, Nottingham | Graham Dow |
| Department of Neurosurgery, Queen's Medical Centre Nottingham, Nottingham | Iain Robertson |
| Department of Neurosurgery, Queen's Medical Centre Nottingham, Nottingham | Laurence Glancz |
| Department of Neurosurgery, Queen's Medical Centre Nottingham, Nottingham | Murugan Sitaraman |
| Department of Neurosurgery, Queen's Medical Centre Nottingham, Nottingham | Ashwin Kumaria |
| Department of Neurosurgery, Queen's Medical Centre Nottingham, Nottingham | Ananyo Bagchi |
| Department of Neurosurgery, John Radcliffe Hospital, Oxford University Hospitals, Oxford | Simon Cudlip |
| Department of Neurosurgery, John Radcliffe Hospital, Oxford University Hospitals, Oxford | Jane Halliday |
| Department of Neurosurgery, John Radcliffe Hospital, Oxford University Hospitals, Oxford | Rory J Piper |
| Department of Neurosurgery, John Radcliffe Hospital, Oxford University Hospitals, Oxford | Alexandros Boukas |
| Department of Neurosurgery, John Radcliffe Hospital, Oxford University Hospitals, Oxford | Meriem Amarouche |
| Department of Neurosurgery, John Radcliffe Hospital, Oxford University Hospitals, Oxford | Damjan Veljanoski |
| Department of Neurosurgery, University Hospitals Plymouth, Plymouth | Sam Muquit |
| Department of Neurosurgery, University Hospitals Plymouth, Plymouth | Ellie Edlmann |
| Department of Neurosurgery, University Hospitals Plymouth, Plymouth | Haritha Maripi |
| Department of Neurosurgery, University Hospitals Plymouth, Plymouth | Yi Wang |
| Department of Neurosurgery, University Hospitals Plymouth, Plymouth | Mehnaz Hossain |
| Department of Neurosurgery, Lancashire Teaching Hospitals NHS Foundation Trust, Preston | Andrew Alalade |
| Department of Neurosurgery, Lancashire Teaching Hospitals NHS Foundation Trust, Preston | Syed Maroof |
| Department of Neurosurgery, Lancashire Teaching Hospitals NHS Foundation Trust, Preston | Pradnya Patkar |
| Department of Neurosurgery, Royal Hallamshire Hospital & Sheffield Children’s Hospital, Sheffield | Saurabh Sinha |
| Department of Otorhinolaryngology, Royal Hallamshire Hospital & Sheffield Children’s Hospital, Sheffield | Showkat Mirza |
| Department of Neurosurgery, Royal Hallamshire Hospital & Sheffield Children’s Hospital, Sheffield | Duncan Henderson |
| Department of Neurosurgery, Royal Hallamshire Hospital & Sheffield Children’s Hospital, Sheffield | Mohammad Saud Khan |
| Department of Neurosurgery, University Hospital Southampton, Southampton | Nijaguna Mathad |
| Department of Neurosurgery, University Hospital Southampton, Southampton | Jonathan Hempenstall |
| Department of Neurosurgery, University Hospital Southampton, Southampton | Difei Wang |
| Department of Neurosurgery, University Hospital Southampton, Southampton | Pavan Marwaha |
| Department of Neurosurgery, Royal Stoke University Hospital, Stoke | Simon Shaw |
| Department of Neurosurgery, Royal Stoke University Hospital, Stoke | Georgios Solomou |
| Department of Neurosurgery, Royal Stoke University Hospital, Stoke | Alina Shrestha |

**Table 2:** List of CRANIAL collaborators (via data validation)

| **Team** | **Name** |
| --- | --- |
| Department of Neurosurgery, Aberdeen Royal Infirmary, Aberdeen | Andrew Fraser |
| Department of Neurosurgery, Royal Victoria Hospital, Belfast | Theodore Hirst |
| Department of Neurosurgery, Queen Elizabeth Hospital Birmingham, Birmingham | Yasir Chowdhury |
| Department of Neurosurgery, Hurstwood Park Neurosciences Centre and Royal Sussex County Hospital, Brighton | Sobiya Bilal |
| Department of Neurosurgery, Southmead Hospital Bristol, Bristol | Jack Wildman |
| Division of Neurosurgery, Cambridge University Hospitals Trust, Cambridge | Ashwin Venkatesh |
| Department of Neurosurgery, University Hospital of Wales, Cardiff | Priya Babu |
| Department of Neurosurgery, Cork University Hospitals, Ireland | Cian Carey |
| Department of Neurosurgery, National Neurosurgical Centre, Beaumont Hospital, Ireland | Renitha Reddi Bathuni |
| Department of Neurosurgery, Ninewells Hospital, Dundee | Kismet Hossain-Ibrahim |
| Department of Neurosurgery, The Western General Hospital, Edinburgh | Joseph Nathaniel Brennan |
| Department of Neurosurgery, Hull University Teaching Hospitals, Hull | Anna Bjornson |
| Department of Neurosurgery, Leeds Teaching Hospitals, Leeds | Howra Ktayen |
| Department of Neurosurgery, The Walton Centre, Liverpool | Sandhya T Trichinopoly |
| Department of Neurosurgery, Barts and The Royal London Hospital, London | Samir Matloob |
| Department of Neurosurgery, Charing Cross Hospital, London | Adarsh Nadig |
| Department of Neurosurgery, King's College Hospital, London | Mohamed Okasha |
| Department of Neurosurgery, National Hospital for Neurology and Neurosurgery, London | Danyal Khan |
| Department of Neurosurgery, Barking, Havering & Redbridge University Hospitals, London | Alireza Shoakazemi |
| Department of Neurosurgery, St George’s University Hospitals Trust, London | Florence Hogg |
| Department of Neurosurgery, Salford Royal Trust, Manchester | Seun Sobawale |
| Department of Neurosurgery, Royal Victoria Infirmary, Newcastle | Amir Suliman |
| Department of Neurosurgery, Queen's Medical Centre Nottingham, Nottingham | Ashwin Kumaria |
| Department of Neurosurgery, John Radcliffe Hospital, Oxford University Hospitals, Oxford | Rory Piper |
| Department of Neurosurgery, John Radcliffe Hospital, Oxford University Hospitals, Oxford | Will Owen |
| Department of Neurosurgery, University Hospitals Plymouth, Plymouth | Ellie Edlmann |
| Department of Neurosurgery, Lancashire Teaching Hospitals NHS Foundation Trust, Preston | Afaq Sartaj |
| Department of Neurosurgery, Royal Hallamshire Hospital & Sheffield Children’s Hospital, Sheffield | Edward Goacher |
| Department of Neurosurgery, University Hospital Southampton, Southampton | Euan Strachan |
| Department of Neurosurgery, Royal Stoke University Hospital, Stoke | Giorgios Solomou |

**Table 3:** Distribution details of variables (predictors, approach, outcome) split by fold. All variables are binary, excluding age which is continuous. For the binary variables the number of entries where the variable is present (represented as a 1) is given. For the singular continuous parameter (age), median; and inter-quartile range (IQR) are given instead.

| **Fold** | **Fold 1** | **Fold 2** | **Fold 3** | **Fold 4** | **Fold 5** | **All** |
| --- | --- | --- | --- | --- | --- | --- |
| *Participants* | 174 | 174 | 173 | 172 | 172 | 865 |
| *EEA Approach* | 028 | 028 | 028 | 028 | 028 | 140 |
| *Median Age (IQR* | 54 (41-64) years | 51 (37-66) years | 54 (42-64) years | 53 (41-63) years | 51 (39-61) years | 53 (40-63) years |
| *Male Sex* | 080 | 086 | 083 | 092 | 075 | 416 |
| *BMI > 30* | 042 | 052 | 049 | 055 | 040 | 238 |
| *Tumour Diameter ≥ 1cm* | 151 | 149 | 146 | 152 | 139 | 737 |
| *Revision Surgery* | 022 | 032 | 022 | 019 | 024 | 119 |
| *Presence of Intraoperative CSF Leak* | 055 | 060 | 061 | 059 | 058 | 293 |
| *CSF Diversion* | 014 | 010 | 017 | 015 | 011 | 067 |
| *Dural Closure* | 000 | 000 | 000 | 000 | 000 | 000 |
| *Dural Replacement* | 050 | 053 | 061 | 050 | 048 | 262 |
| *Vascularised Flap* | 036 | 037 | 048 | 053 | 032 | 206 |
| *Tissue Graft* | 048 | 062 | 056 | 066 | 054 | 286 |
| *Synthetic Graft* | 052 | 039 | 054 | 059 | 046 | 250 |
| *Tissue Glue* | 112 | 115 | 123 | 120 | 117 | 587 |
| *Haemostatic Agent* | 116 | 107 | 102 | 098 | 109 | 532 |
| *Rigid Buttress* | 008 | 008 | 014 | 009 | 009 | 048 |
| *Gasket Seal* | 005 | 004 | 007 | 004 | 006 | 026 |
| *Nasal Packing* | 128 | 137 | 122 | 125 | 122 | 634 |
| *CSFR* | 008 | 008 | 008 | 007 | 007 | 038 |

**Table 4:** SHAP score correlation coefficient for each predictor across all ten NNs. SHAP scores are mean-averaged across the 5-folds and the standard deviations are given after the ‘±’ sign. All values are given to two significant figures.

| Predictor | TSA | EEA | TSA or EEA | TSA | EEA | TSA or EEA | TSA | EEA | TSA or EEA | TSA or EEA |
| --- | --- | --- | --- | --- | --- | --- | --- | --- | --- | --- |
|  | Risk Factors | Risk Factors | Risk Factors | Repair Techniques | Repair Techniques | Repair Techniques | Risk Factors  and  Repair  Techniques | Risk Factors  and  Repair  Techniques | Risk Factors  and  Repair  Techniques | All (including Approach) |
| Age | -0.86±0.06 | -0.06±0.62 | -0.73±0.15 | - | - | - | **-0.77±0.07** | -0.45±0.66 | -0.7±0.25 | -0.77±0.14 |
| Male Sex | 0.64±0.33 | 0.27±0.8 | 0.51±0.53 | - | - | - | 0.47±0.65 | 0.17±0.60 | 0.79±0.12 | 0.68±0.21 |
| BMI > 30 | 0.47±0.47 | 0.27±0.8 | 0.48±0.4 | - | - | - | 0.43±0.70 | -0.4±0.62 | 0.46±0.36 | 0.55±0.28 |
| Tumour Diameter ≥ 1cm | -0.77±0.15 | 0.74±0.21 | 0.33±0.51 | - | - | - | 0.04±0.64 | 0.12±0.94 | 0.28±0.57 | -0.07±0.64 |
| Revision Surgery | 0.63±0.27 | -0.51±0.82 | -0.30±0.56 | - | - | - | 0.17±0.59 | -0.44±0.77 | -0.10±0.57 | 0.27±0.35 |
| Presence of Intraoperative CSF Leak | 0.84±0.04 | 0.00±0.87 | 0.80±0.11 | - | - | - | **0.86±0.04** | 0.43±0.63 | 0.80±0.08 | 0.77±0.10 |
| CSF Diversion | - | - | - | -0.49±0.67 | -0.60±0.46 | -0.8±0.14 | **-0.86±0.04** | **-0.72±0.24** | -0.68±0.37 | -0.83±0.06 |
| Dural Closure | - | - | - | 0.00±0.00 | 0.00±0.00 | 0.00±0.00 | 0.00±0.00 | 0.00±0.00 | 0.00±0.00 | 0.00±0.00 |
| Dural Replacement | - | - | - | 0.64±0.29 | -0.19±0.85 | 0.71±0.22 | 0.49±0.53 | 0.36±0.62 | 0.83±0.07 | 0.54±0.29 |
| Vascularised Flap | - | - | - | 0.28±0.77 | 0.81±0.08 | 0.13±0.84 | **-0.71±0.23** | **0.77±0.18** | 0.26±0.57 | -0.1±0.67 |
| Tissue Graft | - | - | - | 0.77±0.27 | -0.49±0.79 | 0.66±0.45 | 0.52±0.71 | -0.33±0.82 | 0.79±0.08 | 0.63±0.28 |
| Synthetic Graft | - | - | - | -0.05±0.89 | 0.46±0.71 | 0.44±0.40 | **0.77±0.11** | **0.73±0.23** | 0.70±0.10 | 0.77±0.09 |
| Tissue Glue | - | - | - | -0.80±0.14 | -0.54±0.72 | -0.68±0.24 | **-0.77±0.06** | -0.33±0.74 | -0.77±0.08 | -0.80±0.06 |
| Haemostatic Agent | - | - | - | 0.43±0.61 | -0.87±0.07 | 0.28±0.38 | 0.52±0.52 | -0.56±0.64 | 0.32±0.46 | 0.46±0.57 |
| Rigid Buttress | - | - | - | -0.9±0.05 | -0.23±0.83 | -0.61±0.69 | **-0.88±0.07** | **0.78±0.12** | -0.43±0.62 | -0.81±0.03 |
| Gasket Seal | - | - | - | -0.64±0.72 | -0.81±0.34 | -0.91±0.07 | **-0.96±0.01** | **-0.79±0.2** | -0.85±0.11 | -0.92±0.03 |
| Nasal Packing | - | - | - | 0.70±0.42 | 0.85±0.12 | 0.71±0.25 | 0.24±0.71 | 0.53±0.71 | 0.72±0.13 | 0.45±0.61 |
| Surgical Approach | - | - | - | - | - | - | - | - | - | 0.71±0.23 |

**Figure 1:** SHAP scores for predictors displayed as a bee diagram where the NNs are split by both prediction category and approach. Scores are shown for each predictor across all 5-folds. As shown in the ‘predictor value’ legend – a high value is indicated in red, and a low value is indicated by blue; for binary variables this means red indicates a value of 1 (i.e. present) and blue indicates a value of 0 (i.e. not present).


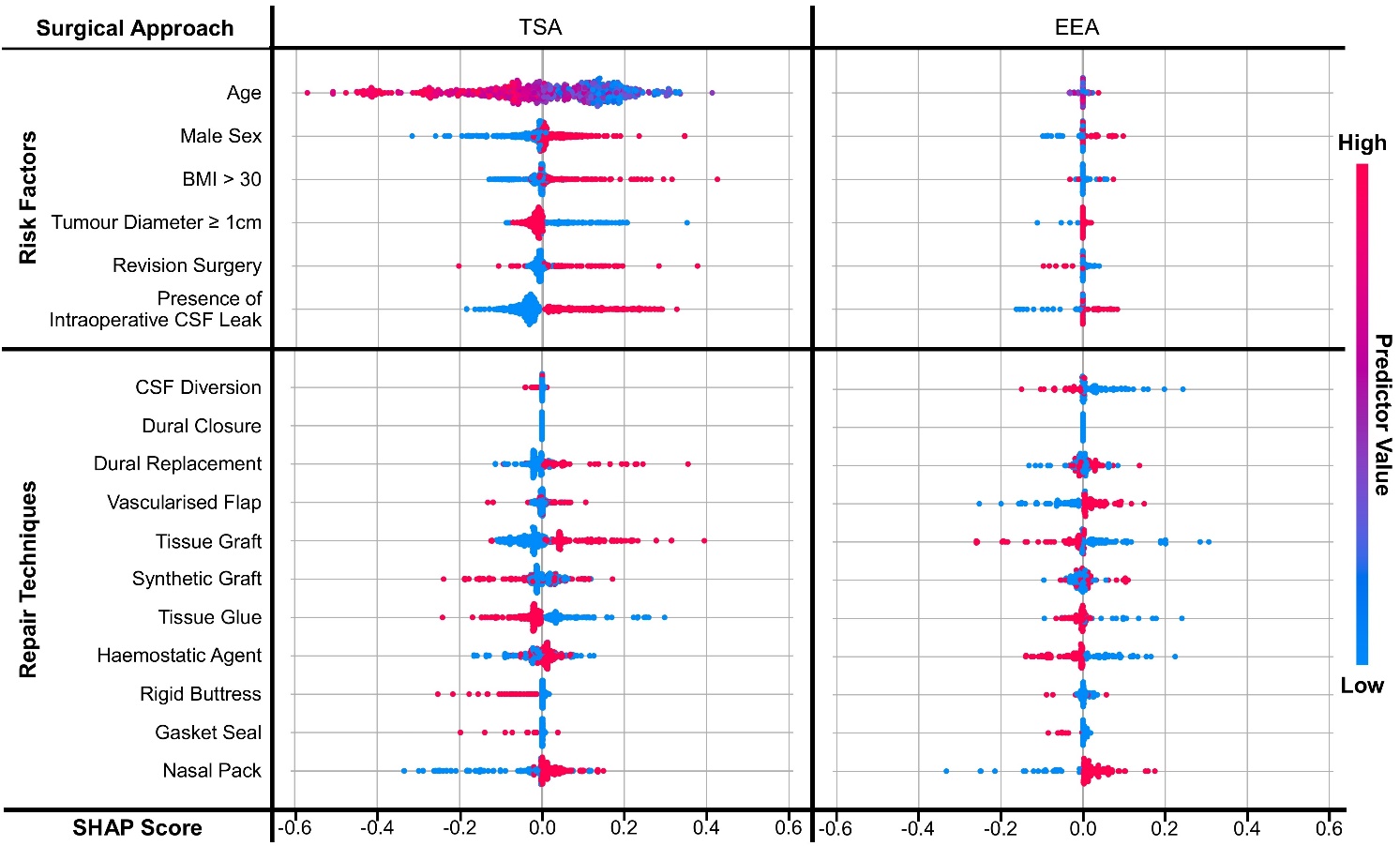


**Figure 2:** SHAP scores for predictors displayed as a bee diagram for the approach category ‘TSA or EEA’, where the NNs are split by prediction category. Scores are shown for each predictor across all 5-folds. As shown in the ‘predictor value’ legend – a high value is indicated in red, and a low value is indicated by blue; for binary variables this means red indicates a value of 1 (i.e. present) and blue indicates a value of 0 (i.e. not present).

**
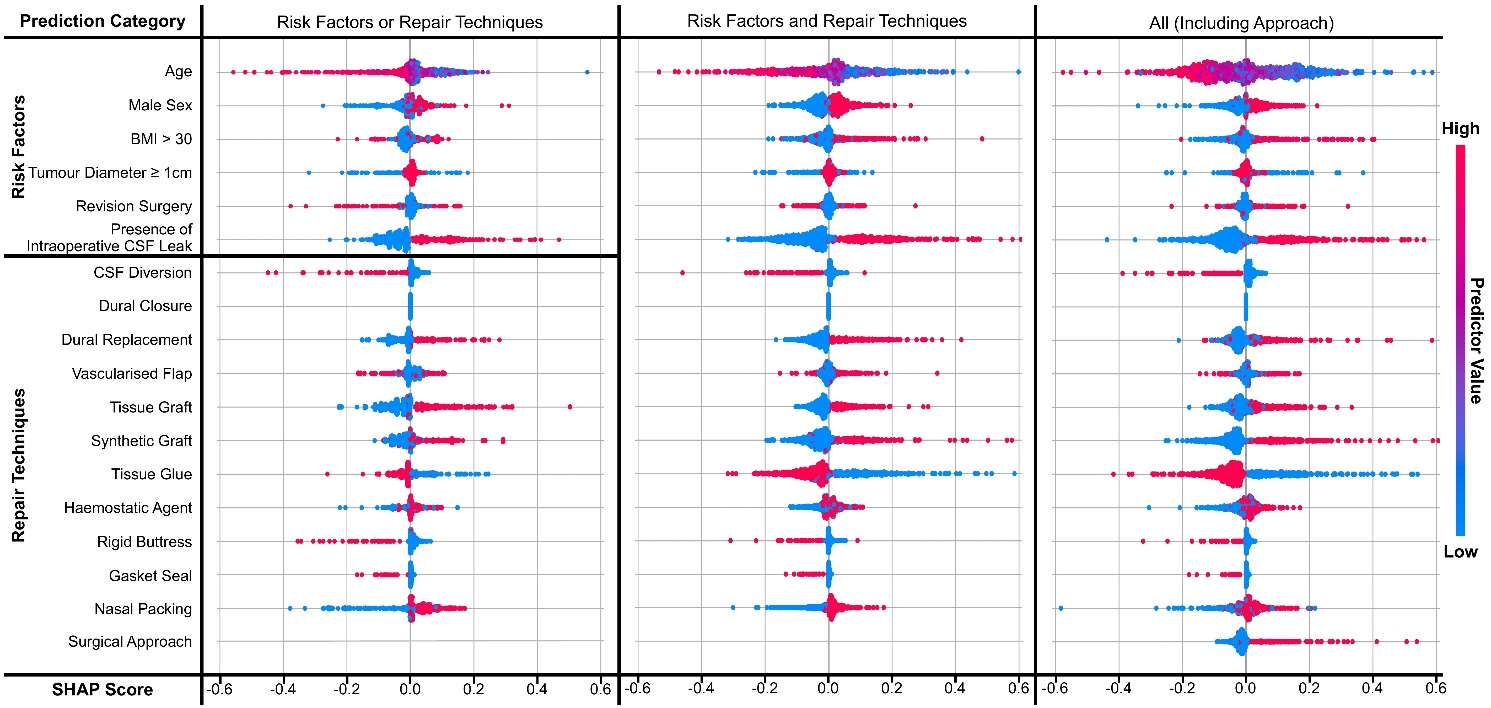
**
